# A novel sensitive hexaplex high-resolution melt assay for identification of five human *Plasmodium* species plus internal control

**DOI:** 10.1016/j.actatropica.2023.107020

**Published:** 2023-12

**Authors:** Suttipat Srisutham, Paweesuda Rattanakoch, Kaewkanha Kijprasong, Rungniran Sugaram, Nantanat Kantaratanakul, Theerarak Srinulgray, Arjen M Dondorp, Mallika Imwong

**Affiliations:** a Department of Clinical Microscopy, Faculty of Allied Health Sciences, Chulalongkorn University, Bangkok, Thailand; b Satan Prabaramee Hospital, Naung Proe, Kanchanaburi, Thailand; c Division of Vector Borne Diseases, Department of Disease Control, Ministry of Public Health, Nonthaburi, Thailand; d Talenome DNA Professional Ltd, Bangkok, Thailand; e Mahidol-Oxford Tropical Medicine Research Unit, Faculty of Tropical Medicine, Mahidol University, Bangkok, Thailand; f Centre for Tropical Medicine and Global Health, Nuffield Department of Medicine, University of Oxford, Oxford, Northern Ireland UK; g Department of Molecular Tropical Medicine and Genetics, Faculty of Tropical Medicine, Mahidol University, Bangkok, Thailand

**Keywords:** Malaria, Detection, PCR, HRM, *Plasmodium*

## Abstract

**Background:**

The diagnosis of malaria infection in humans remains challenging, further complicated by mixed *Plasmodium* species infections, potentially altering disease severity and morbidity. To facilitate appropriate control measures and treatment, rapid, sensitive, and specific detection assays, including those for the second minor species, would be required. This study aimed to develop a multiplex high-resolution melting (hexaplex PCR-HRM) assay with seven distinct peaks corresponding to five *Plasmodium* species of the *Plasmodium* genus, and an internal control to limit false negatives providing quality assurance testing results.

**Methods:**

Five species-specific primers for human malaria species were designed targeting on the *Plasmodium* 18 small subunit ribosomal RNA (18S rRNA) and mitochondrial genes. The hexaplex PCR-HRM was developed for the simultaneous and rapid detection and differentiation of five human *Plasmodium* spp. The limit of detection (LoD), sensitivity, and specificity of the assay were evaluated. Artificial mixing was used to assess the ability to determine the second minor species. Furthermore, a hexaplex PCR-HRM assay was used to identify 120 *Plasmodium*-infected clinical isolates from Kanchanaburi, Western Thailand, where malaria is endemic.

**Results:**

The hexaplex PCR-HRM assay detected the targeted genome of five *Plasmodium* species at levels as low as 2.354–3.316 copies/uL with 91.76 % sensitivity and 98.04 % specificity. In artificial mixing, the assay could detect minority parasite species at 0.001 % of the predominant parasite population. *Plasmodium vivax* infections (99 %) accounted for the majority of malaria cases in Kanchanaburi, Thailand.

**Conclusions:**

The developed hexaplex PCR-HRM assay we present in this study is a novel approach for multiplexing the *Plasmodium* genus and detecting five *Plasmodium* species with the advantage of detecting second minority parasite species. The developed one-step assay without any nesting protocols would reduce the risks of cross-contamination. Moreover, it also provides a simple, sensitive, specific, and low-cost approach for optional molecular detection of malaria.

## Highlights

• Hexaplex high-resolution melt assay for five human *Plasmodium* species were developed.
• The assay detected the genome of five *Plasmodium s*pecies as low as 2.354–3.316 copies/uL.
• The assay could detect minority parasite species at 0.001 % of the population.
• It provides a simple, low-cost approach for optional molecular detection of malaria.

## 1 Background

Malaria infection, caused by the parasite *Plasmodium*, is still a major cause of morbidity and mortality worldwide and reportedly has led to up to 247 million cases and 619,000 deaths in 2021(WHO, 2022). *Plasmodium* parasites that cause malaria in humans include *Plasmodium falciparum, Plasmodium vivax, Plasmodium malariae, Plasmodium ovale*, and *Plasmodium knowlesi* (WHO, 2022). Among these, *P. falciparum* and *P. vivax* are accountable for the majority of malaria cases (WHO, 2022), the former leading to a life-threatening disease (Snow et al., 2005), while the latter being the most widespread malaria parasite globally (Howes et al., 2016; WHO, 2022). Different malaria species require distinct treatment regimens (WHO, 2015). Early and accurate diagnosis to specifically identify the infecting agent among all five malarial species is thus crucial for correct treatment and disease control.

Several methods used for human malaria diagnostics include microscopic examination, rapid diagnostic tests (RDTs), and polymerase chain reaction (PCR). Microscopic examination of *Plasmodium* parasites in stained blood films remains the gold standard but the method is relatively laborious, time-consuming, and requires an experienced analyst (Chilton et al., 2006; Lo et al., 2015). The microscopic detection limit is approximately ∼50–100 parasites/μL. Submicroscopic malaria could also lead to asymptomatic infection misdiagnosis (Kilian et al., 2000). RDTs are often used in addition to microscopic examinations for simple and rapid malaria diagnosis (Wongsrichanalai et al., 2007) but they are not always sufficiently sensitive for detecting low-level parasitemia (Chilton et al., 2006; Orish et al., 2018). PCR-based tests have been extensively described to improve the detection sensitivity and specificity for asymptomatic low parasitemia infections (Tedla, 2019) and are also ideal for diagnosing mixed *Plasmodium* infections (Snounou et al., 1993a). Various PCR-based testing methods have been developed for improving the detection of malaria infection, including nested PCR (nPCR) (Singh et al., 1999; Snounou, 2002; Snounou and Singh, 2002), droplet digital PCR (ddPCR) (Costa et al., 2022; Koepfli et al., 2016; Srisutham et al., 2017), and quantitative real-time PCR (qPCR) (Elsayed et al., 2006; Mangold et al., 2005; Perandin et al., 2004; Rougemont et al., 2004; Shokoples et al., 2009). Nested PCR is considered to be reliable, allowing for a detection limit as low as 1–0.1 parasites/µL of blood (Wang et al., 2014). Although it provides higher sensitivity and specificity than a standard single-tube PCR, the need to transfer the amplicon of the first PCR reaction into the second reaction tube might increase contamination likelihood and analysis time (Abath et al., 2002). Therefore, a single tube reaction assay for specific malaria parasite identification would be preferred, enabling direct closed-tube detection and reducing the time of analysis. qPCR assays for malaria reportedly exhibit high sensitivity and specificity due to their automated, quantitative, and closed-tube system (Monis and Giglio, 2006), representing a highly accurate malaria detection tool to identify asymptomatic low parasitemia infections, important obstacles for malaria elimination efforts (Chua et al., 2015; Swan et al., 2005). qPCR product detection approaches include intercalating dyes (SYBR Green I) and hydrolyze (TaqMan) probes (Zhao et al., 2014). The TaqMan-based detection method is more accurate and reliable but expensive. Meanwhile, intercalating dyes are frequently used as they are cost effective, although they could yield false-positive results from nonspecific amplification (Zhao et al., 2014). In addition, false positive PCR detection has been reported in the case of *P. knowlesi* since its detection primers stochastically cross-react with the genomic DNA of *P. vivax* (Imwong et al., 2009). Multiplex PCR assays have been developed to reduce cost and turnaround time (Chua et al., 2015; Shokoples et al., 2009). Previous studies have reported that multiplexed real-time PCR assays could improve the detection sensitivity for mixed infections (Dormond et al., 2011; Shokoples et al., 2009). However, when co-infecting species were observed mixed infections could still not be detected using multiplex PCR, especially when second species are present in low quantities (Boonma et al., 2007). Therefore, mixed infection identification would be necessary and should be considered in all molecular tests.

Mixed *Plasmodium* species infection could complicate diagnosis and alter disease severity and morbidity. A previous study suggested that *P. vivax* infection over that of *P. falciparum* leads to the rise of *P. falciparum* parasitemia and causes severe malaria. In contrast, *P. falciparum* super infection over an existing *P. vivax* infection reduces *P. falciparum* parasitemia preventing severe malaria development (Mohapatra et al., 2012). Accurate diagnosis of mixed parasite infection would also be important for therapeutic decisions including anti-malarial drug selection, dosage, and timing. The misdiagnosis and mistreatment of single or multiple species have clinical consequences (Lee et al., 2011; Obare et al., 2013).

High-resolution melting (HRM) analysis has been performed to overcome the drawbacks of the intercalating dye method. HRM is a post-PCR analysis to investigate the melting temperature (*T*_m_) of variance in the region of interest within the DNA sequence (Tong and Giffard, 2012). Several qPCR techniques using HRM methods for malaria diagnosis have been proposed to reliably differentiate between mixed malaria infections (Chua et al., 2015; Joste et al., 2018; Kipanga et al., 2014; Lamien-Meda et al., 2021, 2019; Murillo et al., 2019). However, the previous multiplex HRM methods were not designed to include primers specific to the genus *Plasmodium* to confirm malaria infection or reduce false-positive results, and no internal control has been included either to provide assurance that specimens were successfully amplified and detected (Rosenstraus et al., 1998). A previous study suggested that the internal control increases sensitivity by enabling the user to identify and retest samples that might inhibit the PCR reaction. A positive internal control result indicates that amplification has occurred and thus provides assurance that negative test results are truly negative. In addition, the internal control also allows for monitoring any potential competition between multiple targets in multiplex PCR tests. A negative internal control result in specimens that are positive for one target indicates PCR competition and/or inhibition reducing amplification efficiency (Rosenstraus et al., 1998).

This study aimed at developing a hexaplex PCR-HRM assay to detect all five *Plasmodium* species that cause malaria in humans. The assay included a genus-specific target to confirm the positive results and internal controls to reduce false-negative results. The primers were novel and designed for hexaplex PCR-HRM assays based on the 18S rRNA and the mitochondrial cytochrome b genes. We evaluated the limit of detection (LoD), sensitivity, and specificity. DNA from *P. falciparum* and *P. vivax* samples were artificially mixed in various quantities at ratios of 1:1, 1:10, 1:100, and 1:1000 to test the capacity and sensitivity of the assay to identify mixed infections. In addition, the novel hexaplex PCR-HRM was applied to clinical malaria cases.

## 2 Methods

### 2.1 Primer design

The hexaplex PCR-HRM primers were designed using the nucleotide sequences from NCBI databases (https://www.ncbi.nlm.nih.gov/) (Supplementary file 1). The primers were designed based on the genus- and the species-specific nucleotide sequences of the *Plasmodium* 18 small subunit ribosomal RNA (*18S rRNA*) gene for *Plasmodium* spp. and *P. malariae*, as well as the mitochondrial cytochrome b gene for *P. falciparum, P. vivax, P. ovale,* and *P. knowlesi* (Table 1). The primers specific to *P. ovule* were designed to amplify both *P. ovale wallikeri* and *P. ovale curtisi*. The specific and conserved region of each target gene was identified after alignment using the ClustalW algorithm, as implemented in the BioEdit software version 7.2.5. All conserved regions were selected and designed for the hexaplex PCR-HRM primers. All primer pairs were then assessed for specificity via the Basic Local Alignment Sequence Tool (NCBI-BLAST). In addition, the Multiple Primer Analyzer online software (https://www.thermofisher.com) was used to assess the potential of self-dimer and cross-primer dimer formation.

**Table 1:** Primers designed and used for hexaplex PCR-HRM assay.

Primer name	Gene	Sequence (5′–3′)
Falciparum-F	*Cytochrome b*	TCGATAATCACGGGTCGCGGGTTAGGCGTAACTGCTTTCGTTGGTTATATC
Falciparum-R		CGATTGTAAGCACGATCGCCCGTTGCGCGTGCGGTACAATACATAATCCAATAAATGGTGAG
Vivax-F	*Cytochrome b*	TCGATAATCACGGGTCGCGGGTTAGGCGCGTGCGGCGAATATTCAGTACCAATGATATGGCTC
Vivax-R		CGATTGTAAGCACGATCGCCCGTTGCGTGCGGCGGCCATATAAAATAAAAATATCTTGTGGTGAC
Malariae-F	*18* *s rRNA*	TCGATAATCACGGGTCGCGGGTTAATTTATTTATTGAAATTCTTAGATTTTCTGGAGACGAT
Malariae-R		CGATTGTAAGCACGATCGCCCGTTCTCATAAGGTACTGAAGGAAACT
Ovale-F	*Cytochrome b*	TCGATAATCACGGGTCGCGGGTTACTAATTTATTATCTTCAATTCCAACC
Ovale-R		CGATTGTAAGCACGATCGCCCGTTCAATACATAATGCAACRAATGGAGGA
Knowlesi-F	*Cytochrome b*	TCGATAATCACGGGTCGCGGGTTATATTTATTATTAAGTTATTGGGGTGCAACTATC
Knowlesi-R		CGATTGTAAGCACGATCGCCCGTTATATTAATAATAATGTATATCCTCCACATAACCAAGTG
Plasmodium spp-F	*18s rRNA*	TCGATAATCACGGGTCGCGGGTTAGCCGTGCGGCGGCTTAATTTGACTCAACACGGG
Plasmodium spp-R		CGATTGTAAGCACGATCGCCCGTTGCCGTGCGGCCGAAAAACGGCCATGCATCACCAT
PCR-PCT-F	–	TCGATAATCACGGGTCGCGGGTTAGTGCGCTCACCAAGGCGACGATCGGTA
PCR-PCT-R	–	CGATTGTAAGCACGATCGCCCGTTCAATCCGAAGACCTTCATCGTTCAC
Universal forward	–	TCGATAATCACGGGTCGCGGGTTA
Universal reverse	–	CGATTGTAAGCACGATCGCCCGTT

### 2.2 Samples and DNA extraction

To validate the sensitivity and specificity, achieved clinical samples confirmed *Plasmodium* species (*N* = 85) and the samples collected from healthy participants (*N* = 102). The achieved samples with confirmed *Plasmodium* species included *P. falciparum* (*N* = 20), *P. vivax* (*N* = 30), *P. ovale* (*N* = 10) including *P. ovale wallikeri* (*N* = 5) and *P. ovale curtisi* (*N* = 5), *P. malariae* (*N* = 10), and *P. knowlesi* (*N* = 15) obtained from a previous study, describing a malaria infection outbreak at Ubon Ratchathani, Northeastern Thailand, in 2014 (Imwong et al., 2014) and at Chumphon, Southern Thailand in 2018 and 2019 (Sugaram et al., 2021). These samples have been obtained from the ethical review committees of the Faculty of Tropical Medicine, Mahidol University (approval no. MUTM 2012-045-05, MUTM2020-082-01). In addition, the blood samples were collected from symptomatic patients with malaria diagnosed by peripheral blood microscopy in Kanchanaburi, Thailand (*N* = 120). Ethical approvals for the study were obtained from the ethical review committees of Chulalongkorn University (approval no. COA 010/66). DNA samples were extracted from 200 μL of the EDTA blood sample using the DNA isolation kit (macrogen, Germany), following the manufacturer's instructions.

To validate the hexaplex PCR-HRM approach, we produced and used plasmid DNA in this study. The constructed plasmid DNA (BBI Life Sciences Corporation, China) was used under optimized PCR-HRM conditions in a real-time PCR thermocycler machine (Hangzhou Bio-Gener Technology Co., Ltd). The plasmids were also used in serial dilutions from 100,000 to 0.1 copies/µL to estimate the detection limit.

### 2.3 DNA amplification and HRM analysis

**Fig. 1:**
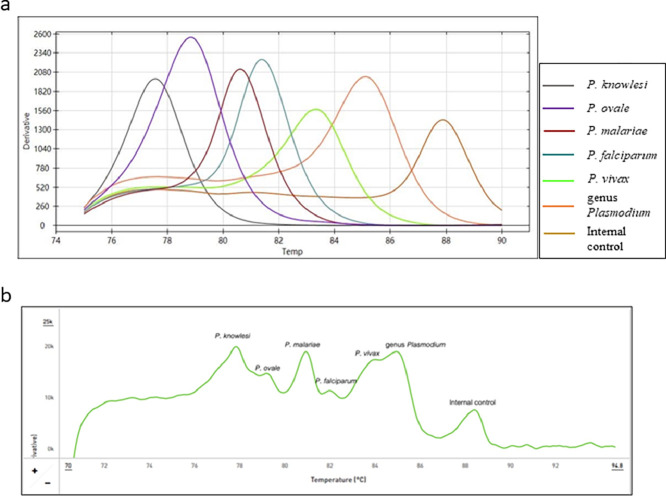
Multiplex HRM melting curve in derivative plot analyses. Merged wells analysis showing separate peaks for *Plasmodium* species using plasmid DNA of five *Plasmodium* species (a). Specific peaks using a plasmid DNA mix of five *Plasmodium* species (b).

The hexaplex PCR-HRM reaction was performed in a total volume of 25 µL, containing 0.64× Hot Start PCR Master mix (Apsalagen Co.Ltd., Thailand), 1.25× Midori dye (Nippon Genetics Co.Ltd., Japan) dye for detecting double stranded DNA in the HRM analysis, 115 nM of each primer pair specific to *P. ovale, P. malariae, P. vivax*, or *P. knowlesi*, 70 nM of primer pair specific to *P. falciparum*, 270 nM of primer specific to the genus *Plasmodium*, 70 nM of primer pair specific to internal control, 20 nM of universal forward and reverse primers (Bionics Co.Ltd, Korea), 3 µL of extract DNA template, and 100,000 copies of plasmid DNA template as an internal PCR control. The PCR was performed using the following cycling protocol: initial denaturation step at 95 °C for 5 min followed by 37 cycles at 95 °C for 30 s, 57 °C for 45 s, 72 °C for 30 s, then 13 cycles at 95 °C for 30 s, 65 °C for 45 s, 72 °C for 30 s, and a final extension step at 72 °C for 2 min. The HRM temperature was raised from 75 °C to 95 °C. During this process, the amplicons obtained from the previous PCR were denatured prior to the development of the melting curves in the inflection point where the changes in fluorescence with respect to the changes in temperature (Df/Dt) were recorded with a ramp of 0.3 °C/s (9) and the fluorescence dye signaling was measured after each cycle. The HRM analysis in the real-time PCR machine was used for the analysis. Fig. 1a presents the Tm of the multiplex reaction.

### 2.4 Hexaplex PCR-HRM assay validation

The developed hexaplex PCR-HRM assay was validated for analytical sensitivity (lower LoD) using the plasmid DNA at concentrations of 100,000, 10,000, 1000, 100, 10, 1, and 0.1 copies/µL. Each standard plasmid DNA sample test was performed in 4 replicates. The analytical sensitivity was determined by probit analysis using SPSS 21.0.

The sensitivity and specificity of the developed hexaplex PCR-HRM assay were analyzed using the genomic DNA extracted from patients with malaria and the species were confirmed using real-time PCR assay as a reference method. The real-time PCR assays were performed as described previously (Perandin et al., 2004; Rougemont et al., 2004). A total of 10 μL per reaction contained 400 nM of the forward and reverse primers and 200 nM of the probe, at an annealing temperature of 60 °C for *P. falciparum* and *P. vivax* detection and an annealing temperature of 52 °C for detecting *P. malariae* and *P. ovale*. We interpreted the result as positive when the Ct-value of the real-time PCR was lower than 40. For *P. knowlesi*, the species identification was analyzed using the sequencing of previous protocols (Snounou et al., 1993b; Sugaram et al., 2021).

The artificial mix of *P. falciparum* and *P. vivax* was prepared by mixing *P. falciparum* with *P. vivax* collected from Kanchanaburi in different ratios. The number of parasites was measured based on the following formula: number of parasites/µL = number of parasites counted ×  8000 WBC count/μL)/ number of WBC counted. To test the capacity of the assay to identify mixed infections the following ratios have been used: 1:1, 1:10, 1:100, and 1:1000.

## 3 Results

### 3.1 Hexaplex PCR-HRM development

The hexaplex PCR-HRM was designed to provide different Tms between the genus and species (Table 2 and Fig. 1a). The *P. falciparum-, P. vivax-, P. ovale-, P. malariae-, P. knowlesi-,* and genus-specific Tms were 81.8 °C, 83.8 °C, 79.3 °C, 81.0 °C, 78.0 °C, and 84.5 °C, respectively (Table 2).

**Table 2:** Hexaplex PCR-HRM *Plasmodium* species-specific Tm Mean, SD, and CV values.

	*T*_m_ ( °C)					
	*P. falciparum*	*P. vivax*	*P. ovale*	*P. malariae*	*P. knowlesi*	Genus
Mean	81.8	83.8	79.3	81.0	78.0	84.5
SD	0.29	0.26	0.26	0.13	0.13	0.56
%CV	0.36	0.31	0.32	0.15	0.16	0.66

### 3.2 Hexaplex PCR-HRM validation

**Fig. 2:**
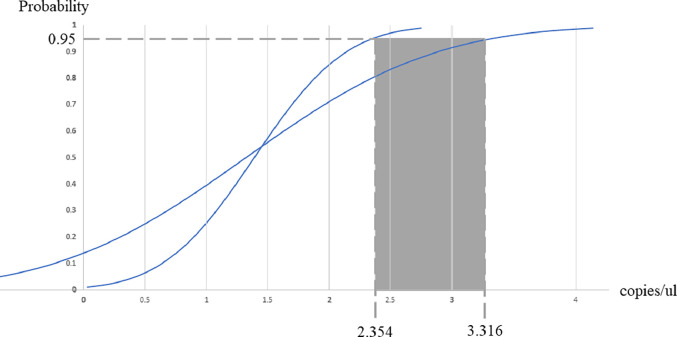
Probit analysis showing 95 % probability to detect five human malaria-related *Plasmodium* species.

Concerning the analytical sensitivity based on the probit analysis, the threshold for 95 % of parasite genome detection probability using the hexaplex PCR-HRM assay was as low as 2.4–3.3 copies/µL (Fig. 2 and Supplementary file 2). The hexaplex PCR-HRM assay provides 91.76 % sensitivity and 98.04 % specificity to detect and identify the five *Plasmodium* species-related malaria infections using the real-time PCR assay described previously as the reference method for *P. falciparum, P. vivax, P. ovale*, and *P. malariae* identification (Perandin et al., 2004; Rougemont et al., 2004), and DNA sequencing reference methods from other sources (Snounou et al., 1993b; Sugaram et al., 2021) for *P. knowlesi* identification (Supplementary file 3). To demonstrate the effect of the PCR competition of the hexaplex PCR-HRM assay, we prepared a plasmid DNA mix of the five *Plasmodium* species and the hexaplex PCR-HRM assay could detect all species-specific peaks of the mix, which limited the PCR competition effect (Supplementary file 1b). In addition, to test the capacity of the assay to identify mixed infections, the hexaplex PCR-HRM assay was tested in an artificial mix of *P. falciparum* and *P. vivax* at ratios of 1:1, 1:10, 1:100, and 1:1000 (Table 3). The assay could detect *P. falciparum* at a ratio of 1:1000 relative to *P. vivax* and could also detect *P. vivax* as a minor species against *P. falciparum.*

**Table 3:** *Plasmodium* spp. detection and identification in an artificial mix of *P. falciparum* and *P. vivax* using hexaplex PCR-HRM analysis.

Artificial mixed		Hexaplex PCR-HRM results in quadruplicate					
*P. falciparum* (parasites/ul)	*P. vivax* (parasites/ul)	*P. falciparum*	*P. vivax*	*P. malariae*	*P. ovale*	*P. knowlwsi*	*Genus Plasmodium*
1000	1000	+/+/+/+	+/+/+/+	-/-/-/-	-/-/-/-	-/-/-/-	+/+/+/+
1000	100	+/+/+/+	+/+/+/+	-/-/-/-	-/-/-/-	-/-/-/-	+/+/+/+
1000	10	+/+/+/+	+/-/+/-	-/-/-/-	-/-/-/-	-/-/-/-	+/+/+/+
1000	1	+/+/+/+	+/-/+/-	-/-/-/-	-/-/-/-	-/-/-/-	+/+/+/+
1000	1000	+/+/+/+	+/+/+/+	-/-/-/-	-/-/-/-	-/-/-/-	+/+/+/+
100	1000	+/+/+/+	+/+/+/+	-/-/-/-	-/-/-/-	-/-/-/-	+/+/+/+
10	1000	+/+/+/+	+/+/+/+	-/-/-/-	-/-/-/-	-/-/-/-	+/+/+/+
1	1000	-/+/+/-	+/+/+/+	-/-/-/-	-/-/-/-	-/-/-/-	+/+/+/+

### 3.3 Hexaplex PCR-HRM application for *Plasmodium* detection and identification

Using the novel hexaplex PCR-HRM, we detected and identified *Plasmodium* parasites from a total of 120 symptomatic patients with malaria in Kanchanaburi, Thailand, in 2022 and 2023. Our results showed that out of the 120 samples, 99.2 % were caused by *P. vivax*, while *P. falciparum* could be detected in 0.8 % of the clinical isolates (Supplementary file 4). Comparisons of the results obtained using our novel hexaplex PCR-HRM assay and those of microscopic examinations indicated that the results of the two detection methods were in 100 % agreement (κ = 1.00). In addition, based on the clinical samples, the developed hexaplex PCR-HRM allowed for the detection of *P. vivax* at concentrations as low as 99–232 parasites/mL at a probability of 95 % (Supplementary file 4).

## 4 Discussion

To effectively control and eliminate malaria, rapid, sensitive, and specific detection assays would be essential for facilitating appropriate control and treatment approaches. despite their significant sensitivity and specificity, molecular assays have certain limitations, including turnaround time, cost, and the required specialized molecular equipment.

In this study, we present a hexaplex PCR-HRM assay we developed and designed for the detection and differentiation of five *Plasmodium* species causing malaria in humans, including *P. falciparum, P. vivax, P. ovale, P. malariae*, and *P. knowlesi*. For higher sensitivity, the primers were designed to target the 18S rRNA and cytochrome b genes, which obtained multiple copies in the genome (Amaral et al., 2019; Mercereau-Puijalon et al., 2002). The primers were newly designed based on 113 nucleotide sequences of the 18S rRNA and the cytochrome b genes of Plasmodium species to increase sensitivity and specificity. The P. falciparum-specific primers were designed using the cytochrome b gene encoding for amino acids 120–185, which do not overlap with the codon Y268S atovaquone resistance target (Saunders et al., 2015). The hexaplex PCR-HRM assay clearly identified seven melting peaks. The difference of the melting temperature peaks was 0.7–2 °C, enabling clear discrimination between the five Plasmodium species-, genus-, and internal control-specific samples, comparable to a previous qRT-PCR-HRM assay, where, however, the differences between the melting temperature peaks were 0.5–1 °C, making it impossible to discriminate between *P. falciparum* and *P. malariae* (27). In addition, our novel hexaplex PCR-HRM assay also includes an internal control. A synthetic internal control, a plasmid DNA with primer binding regions identical to the target sequence, was introduced into the amplification reaction mixture, enabling us to monitor amplification and detection (Rosenstraus et al., 1998). The positive Plasmodium infection was confirmed by the melting peaks of both the genus-specific and internal controls. The novel universal *Plasmodium* target primers designed and included in the multiplex PCR-HRM assay may be able to detect other *Plasmodium* species that can accidentally infect humans, including *P. cynomolgi, P. brasilianum, P. fragile, P. yoelii, P. gaboni, P. berghei*, and *P. gallinaceum*.

The performance of the novel hexaplex PCR-HRM assay was evaluated and compared with reference methods (Perandin et al., 2004; Rougemont et al., 2004; Snounou et al., 1993b; Sugaram et al., 2021) providing 91.76 % sensitivity and 98.04 % specificity. However, the sample sites of *P. ovale* and *P. malariae* are low since there is a low prevalence of these two *Plasmodium* species infections in Thailand. The developed multiplex HRM assay could detect the target genome of the five *Plasmodium* species at concentrations as low as 2.354–3.316 copies/µL, comparable to a previously developed qPCR-HRM assay that could detect the target sequence of the five *Plasmodium* species until 1–100 copies/µL (Chua et al., 2015), and comparable to a previous qPCR-HRM assay using the TaqMan probe in *Plasmodium* species with the differentiation threshold to detect parasites as low as 21.47–46.34 copies/µL. Therefore, the %CV of the Tm obtained from this study confirming the reproducibility of the assay was very high.

One of the major limitations of previous molecular techniques is the capacity to detect mixed infections (Bialasiewicz et al., 2007; Calderaro et al., 2007; Rougemont et al., 2004) due to the primers that favored the amplification of the fragments that belonged to the species with the highest infection level. To increase the detection sensitivity of the mixed infections, universal primers were designed and used for our hexaplex PCR-HRM assay. In this study, we prepared samples of mixed *P. falciparum* and *P. vivax* infections and tested them using our novel hexaplex PCR-HRM assay. Our results suggested that the assay could successfully detect *P. falciparum* parasites at a ratio of 1:1000 relative to *P. vivax*, and vice versa. The developed hexaplex PCR-HRM assay might thus improve the detection sensitivity of mixed infections compared to previous multiplex real-time PCR assays, which could detect *P. falciparum* at a ratio of 1:100 relative to *P. vivax, P. malariae*, and *P. ovale* (Shokoples et al., 2009).

The turnaround time for performing our newly developed hexaplex PCR-HRM assay was 120  min, including both the amplification and melting curve analysis. This turnaround time is the same as that of a previously developed qPCR-HRM (Chua et al., 2015) but is faster than the conventional nPCR-based approaches with additional PCR product separation by electrophoresis in agarose gel (Fuehrer et al., 2012; Mfuh et al., 2019; Snounou et al., 1993a). The estimated cost of this developed SYBR Green-Based HRM assay is approximately $4 per reaction, which is lower than that of the TaqMan probe HRM assay (Chua et al., 2015).

Using our newly developed multiplex PCR-HRM assay in patients with symptomatic malaria in Kanchanaburi, Thailand, in 2022 and 2023, we found that most malaria cases were caused by *P. vivax.* We could not detect any case of *P. knowlesi*-derived malaria in this study. Therefore, the developed hexaplex PCR-HRM assay presented in this study could be potentially applied for the surveillance and epidemiology-related studies of the described five *Plasmodium* species including *P. knowlesi*. Although we identified no mixed infection in this study, we undertook experiments using an artificial DNA mix from *P. vivax* and *P. falciparum* samples and could detect all the peaks of the mixed species limiting the effect of PCR competition.

## 5 Conclusions

In this study, targeting mitochondrial DNA, we developed and validated a novel hexaplex PCR-HRM for the detection of all the five *Plasmodium* species that cause malaria in humans. The assay we developed is proven to be highly sensitive, specific, cost effective, and easy to perform. This assay could be a useful tool for timely and accurate detection of *Plasmodium* infection supporting malaria elimination and control.

## Ethics approval and consent to participate

Ethical approvals for the study were obtained from the ethical review committees of the Faculty of Tropical Medicine, Mahidol University (approval no. MUTM 2012-045-05, MUTM2020-082-01) and ethical review committees of the Chulalongkorn University (approval no. COA 010/66).

## Consent for publication

Not applicable.

## Availability of data and materials

All data generated or analysed during this study are included in this published article and its supplementary information files.

## Funding

This Research is funded by Thailand Science research and Innovation Fund Chulalongkorn University (HEA663700102), Grants for development of new faculty staff, Ratchadapisek Somphot Fund, Chulalongkorn University. Research funds of Faculty of Allied Health Sciences, Chulalongkorn University. This research project was also supported by 10.13039/501100014571Mahidol University, MU-MRC to MI, and part of the Mahidol-University Oxford Tropical Medicine Research Programme funded by the Wellcome Trust of the United Kingdom (core grant 106698/B/14/Z) and Wellcome OA statement. This research was funded in whole, or in part, by the 10.13039/100010269Wellcome Trust [220211]. For the purpose of Open Access, the author has applied a CC BY public copyright licence to any Author Accepted Manuscript version arising from this submission. The funding sources did not participate in data analysis or the final decision to publish the manuscript.

## Authors' contributions

SS, PR and MI contributed to study design. KK and RS collected samples. SS, PR, NK and TS undertook laboratory work. SS and MI Analysed data. SS, AD and MI drafted the manuscript. All authors read and approved the final manuscript.

## CRediT authorship contribution statement

**Suttipat Srisutham:** Methodology, Validation, Software, Data curation, Formal analysis, Visualization, Writing – original draft. **Paweesuda Rattanakoch:** Methodology, Validation, Software, Data curation, Formal analysis. **Kaewkanha Kijprasong:** Resources. **Rungniran Sugaram:** Resources. **Nantanat Kantaratanakul:** Software, Formal analysis. **Theerarak Srinulgray:** Software, Formal analysis. **Arjen M Dondorp:** Funding acquisition, Writing – review & editing. **Mallika Imwong:** Conceptualization, Investigation, Supervision, Funding acquisition, Writing – review & editing.

## Declaration of Competing Interest

The authors declare that they have no known competing financial interests or personal relationships that could have appeared to influence the work reported in this paper.

## Data Availability

Data will be made available on request.

## Appendix Supplementary materials

Image, application 1
