# Corrigendum to: A novel sensitive hexaplex high-resolution melt assay for identification of five human *Plasmodium* species plus internal control (Acta Tropica, volume 248, article number 107020)

**DOI:** 10.1016/j.actatropica.2023.107077

**Published:** 2024-01

**Authors:** Suttipat Srisutham, Paweesuda Rattanakoch, Kaewkanha Kijprasong, Rungniran Sugaram, Nantanat Kantaratanakul, Theerarak Srinulgray, Arjen M Dondorp, Mallika Imwong

**Affiliations:** a Department of Clinical Microscopy, Faculty of Allied Health Sciences, Chulalongkorn University, Bangkok, Thailand; b Satan Prabaramee Hospital, Naung Proe, Kanchanaburi, Thailand; c Division of Vector Borne Diseases, Department of Disease Control, Ministry of Public Health, Nonthaburi, Thailand; d Talenome DNA Professional Ltd, Bangkok, Thailand; e Mahidol-Oxford Tropical Medicine Research Unit, Faculty of Tropical Medicine, Mahidol University, Bangkok, Thailand; f Centre for Tropical Medicine and Global Health, Nuffield Department of Medicine, University of Oxford, Oxford, UK; g Department of Molecular Tropical Medicine and Genetics, Faculty of Tropical Medicine, Mahidol University, Bangkok, Thailand

The statement "MU-MRC to MI" listed in the funding section of our publication is incorrect and should be revised to "MU's Strategic Research Fund: 2023 to MI." Additionally, the address for the corresponding authors needs to be updated to "Department of Molecular Tropical Medicine and Genetics, Faculty of Tropical Medicine, Mahidol University, Bangkok, Thailand." The updated affiliation is as stated above. The authors would like to apologize for any inconvenience caused.

The authors and the Publisher regret the error that appeared in their paper.

