# Supplementary material for: A novel sensitive hexaplex high-resolution melt assay for identification of five human Plasmodium species plus internal control
Source: Acta Trop. 2023 Dec;248:107020. doi: 10.1016/j.actatropica.2023.107020 (PMC10641754; doi:10.1016/j.actatropica.2023.107020)

Supplementary file 1. Nucleotide sequences used for primers design.

| No. | Gene | *Plasmodium* spp. | Accession Number |
| --- | --- | --- | --- |
| 1 | *18S rRNA* | *Plasmodium falciparum* | XR_002966679.1 |
| 2 | *18S rRNA* | *Plasmodium falciparum* | JQ627152.1 |
| 3 | *18S rRNA* | *Plasmodium falciparum* | MN852862.1 |
| 4 | *18S rRNA* | *Plasmodium vivax* | HQ283224.1 |
| 5 | *18S rRNA* | *Plasmodium vivax* | GU816245.1 |
| 6 | *18S rRNA* | *Plasmodium vivax* | XR_003001211.1 |
| 7 | *18S rRNA* | *Plasmodium vivax* | U93095.1 |
| 8 | *18S rRNA* | *Plasmodium ovale* | KF696369.1 |
| 9 | *18S rRNA* | *Plasmodium ovale* | KY073344.1 |
| 10 | *18S rRNA* | *Plasmodium malariae* | KJ934252.1 |
| 11 | *18S rRNA* | *Plasmodium malariae* | KR105947.1 |
| 12 | *18S rRNA* | *Plasmodium malariae* | KJ934251.1 |
| 13 | *18S rRNA* | *Plasmodiun knowlesi* | XR_005506393.1 |
| 14 | *18S rRNA* | *Plasmodiun knowlesi* | MG570077.1 |
| 15 | *18S rRNA* | *Plasmodiunm gallinaceum* | XR_003699242.1 |
| 16 | *18S rRNA* | *Plasmodium cynomolgi* | DQ660816.1 |
| 17 | *18S rRNA* | *Plasmodium chabaudi* | DQ241815.1 |
| 18 | *18S rRNA* | *Plasmodium reichenowi* | XR_001167188.2 |
| 19 | *18S rRNA* | *Plasmodium brasilianum* | KT266778.1 |
| 20 | *18S rRNA* | *Plasmodium yoelii* | XR_002696559.2 |
| 21 | *18S rRNA* | *Plasmodium berghei* | AJ243513.1 |
| 22 | *18S rRNA* | *Plasmodium fragile* | XR_001111607.1 |
| 23 | *18S rRNA* | *Plasmodium inui* | XR_606809.1 |
| 24 | *18S rRNA* | *Plasmodium gaboni* | XR_001974520.1 |
| 25 | *18S rRNA* | *Plasmodium gallinaceum* | XR_003699198.1 |
| 26 | *18S rRNA* | *Plasmodium coatneyi* | XR_002198253.1 |
| 27 | *18S rRNA* | *Plasmodium sp.* | XR_003699387.1 |
| 28 | *18S rRNA* | *Plasmodium reichenowi* | GU815536.1 |
| 29 | *18S rRNA* | *Plasmodium sp.* | XR_003699303.1 |
| 30 | *18S rRNA* | *Plasmodium gaboni* | XR_001974520.1 |
| 31 | *18S rRNA* | *Plasmodium cynomolgi* | MN368129.1 |
| 32 | *18S rRNA* | *Plasmodium billcollinsi* | GU815537.1 |
| 33 | *18S rRNA* | *Plasmodium fieldi* | FJ619102.1 |
| 34 | *18S rRNA* | *Plasmodium simium* | U69605.1 |
| 35 | *18S rRNA* | *Plasmodium reichenowi* | EU560451.1 |
| 36 | *18S rRNA* | *Plasmodium juxtanucleare* | AF463507.1 |
| 37 | *18S rRNA* | *Plasmodium hylobati* | AY579421.1 |
| 38 | *18S rRNA* | *Plasmodium relictum* | AF145399.1 |
| 39 | *18S rRNA* | *Leucocytozoon sp.* | MK650858.1 |
| 40 | *18S rRNA* | *Hepatocystis sp.* | MK078100.1 |
| 41 | *18S rRNA* | *Plasmodium brasilianum* | KU999995.1 |
| 42 | *18S rRNA* | *Plasmodium brasilianum* | KT266778.1 |
| 43 | *18S rRNA* | *Plasmodium brasilianum* | KJ619941.1 |
| 44 | *cytochrome B* | *Plasmodium falciparum* | LR605957.1 |
| 45 | *cytochrome B* | *Plasmodium falciparum* | MK203840.1 |
| 46 | *cytochrome B* | *Plasmodium falciparum* | LR131495.1 |
| 47 | *cytochrome B* | *Plasmodium falciparum* | MH974140.1 |
| 48 | *cytochrome B* | *Plasmodium falciparu* | NC_037526.1 |
| 49 | *cytochrome B* | *Plasmodium falciparum* | KY923500.1 |
| 50 | *cytochrome B* | *Plasmodium sp.* | KU665685.1 |
| 51 | *cytochrome B* | *Plasmodium sp.* | KT824292.1 |
| 52 | *cytochrome B* | *Plasmodium lomamiensis* | KY790541.1 |
| 53 | *cytochrome B* | *Plasmodium reichenowi* | KU665697.1 |
| 54 | *cytochrome B* | *Plasmodium vinckei* | LR865437.1 |
| 55 | *cytochrome B* | *Plasmodium ovale wallikeri* | MF693443.1 |
| 56 | *cytochrome B* | *Plasmodium ovale wallikeri* | KJ930413.1 |
| 57 | *cytochrome B* | *Plasmodium ovale curtisi* | KP050432.1 |
| 58 | *cytochrome B* | *Plasmodium ovale* | KC175307.1 |
| 59 | *cytochrome B* | *Plasmodium ovale wallikeri* | HQ712053.1 |
| 60 | *cytochrome B* | *Plasmodium ovale* | GU815518.1 |
| 61 | *cytochrome B* | *Plasmodium ovale* | AB354571.1 |
| 62 | *cytochrome B* | *Plasmodium ovale* | LT594520.1 |
| 63 | *cytochrome B* | *Plasmodium malariae* | MF693442.1 |
| 64 | *cytochrome B* | *Plasmodium malariae* | LT594637.1 |
| 65 | *cytochrome B* | *Plasmodium malariae* | KU759826.1 |
| 66 | *cytochrome B* | *Plasmodium malariae* | GU815517.1 |
| 67 | *cytochrome B* | *Plasmodium malariae* | AB489194.1 |
| 68 | *cytochrome B* | *Plasmodium malariae* | AF069624.1 |
| 69 | *cytochrome B* | *Plasmodium knowlesi* | LR701176.1 |
| 70 | *cytochrome B* | *Plasmodium knowlesi* | KU245040.1 |
| 71 | *cytochrome B* | *Plasmodium knowlesi* | KC847052.1 |
| 72 | *cytochrome B* | *Plasmodium knowlesi* | JQ345523.1 |
| 73 | *cytochrome B* | *Plasmodium knowlesi* | JF419323.1 |
| 74 | *cytochrome B* | *Plasmodium knowlesi* | AB444108.1 |
| 75 | *cytochrome B* | *Plasmodium knowlesi* | EU880498.1 |
| 76 | *cytochrome B* | *Plasmodium vivax* | KY012318.1 |
| 77 | *cytochrome B* | *Plasmodium vivax* | KY923424.1 |
| 78 | *cytochrome B* | *Plasmodium vivax* | KY923423.1 |
| 79 | *cytochrome B* | *Plasmodium vivax* | KY923387.1 |
| 80 | *cytochrome B* | *Plasmodium vivax* | KY923373.1 |
| 81 | *cytochrome B* | *Plasmodium vinckei* | LR865437.1 |
| 82 | *cytochrome B* | *Plasmodium vinckei vinckei* | AB599931.1 |
| 83 | *cytochrome B* | *Plasmodium vinckei petteri* | DQ414656.1 |
| 84 | *cytochrome B* | *Plasmodium vinckei vinckei* | LR215072.1 |
| 85 | *cytochrome B* | *Plasmodium brasilianum* | KY709306.1 |
| 86 | *cytochrome B* | *Plasmodium brasilianum* | GQ355484.1 |
| 87 | *cytochrome B* | *Plasmodium yoelii yoelii* | MK395263.1 |
| 88 | *cytochrome B* | *Plasmodium yoelii* | KU318044.1 |
| 89 | *cytochrome B* | *Plasmodium yoelii* | LK934644.1 |
| 90 | *cytochrome B* | *Plasmodium yoelii* | EU254521.1 |
| 91 | *cytochrome B* | *Plasmodium yoelii nigeriensis* | DQ414659.1 |
| 92 | *cytochrome B* | *Plasmodium yoelii* | AY099051.1 |
| 93 | *cytochrome B* | *Plasmodium berghei* | XM_034564809.1 |
| 94 | *cytochrome B* | *Plasmodium berghei* | XM_034567869.1 |
| 95 | *cytochrome B* | *Plasmodium berghei* | LC541748.1 |
| 96 | *cytochrome B* | *Plasmodium berghei* | XM_034567065.1 |
| 97 | *cytochrome B* | *Plasmodium chabaudi adami* | AB379671.1 |
| 98 | *cytochrome B* | *Plasmodium chabaudi adami* | DQ414648.1 |
| 99 | *cytochrome B* | *Plasmodium chabaudi adami* | AB379670.1 |
| 100 | *cytochrome B* | *Plasmodium chabaudi* | EU254523.1 |
| 101 | *cytochrome B* | *Plasmodium reichenowi* | KU665697.1 |
| 102 | *cytochrome B* | *Plasmodium reichenowi* | KY790524.1 |
| 103 | *cytochrome B* | *Plasmodium reichenowi* | KU665669.1 |
| 104 | *cytochrome B* | *Plasmodium nucleophilum* | JX467689.1 |
| 105 | *cytochrome B* | *Plasmodium gaboni* | KU759806.1 |
| 106 | *cytochrome B* | *Plasmodium gallinaceum* | LC506179.1 |
| 107 | *cytochrome B* | *Plasmodium gallinaceum* | LN835294.1 |
| 108 | *cytochrome B* | *Plasmodium gallinaceum* | KP025675.1 |
| 109 | *cytochrome B* | *Plasmodium gallinaceum* | AB599930.1 |
| 110 | *cytochrome B* | *Plasmodium inui* | MH974133.1 |
| 111 | *cytochrome B* | *Plasmodium inui* | KJ569850.1 |
| 112 | *cytochrome B* | *Plasmodium inui* | AB444112.1 |
| 113 | *cytochrome B* | *Plasmodium inui* | GQ355483.1 |

**Supplementary file 2. Hexaplex PCR-HRM assay analytical sensitivity**

*Plasmodium falciparum*

| Cell Counts and Residuals | | | | | | | |
| --- | --- | --- | --- | --- | --- | --- | --- |
| PROBIT | Number | Concentration | Number of Subjects | Observed Responses | Expected Responses | Residual | Probability |
|  | 1 | 100000 | 4 | 4 | 4 | 0 | 1 |
|  | 2 | 10000 | 4 | 4 | 4 | 0 | 1 |
|  | 3 | 1000 | 4 | 4 | 4 | 0 | 1 |
|  | 4 | 100 | 4 | 4 | 4 | 0 | 1 |
|  | 5 | 10 | 4 | 4 | 4 | 0 | 1 |
|  | 6 | 1 | 4 | 1 | 1.013 | -0.013 | 0.253 |
|  | 7 | 0.1 | 4 | 0 | 0.056 | -0.056 | 0.014 |

| Probability | Estimate |
| --- | --- |
| 0.01 | 0.026 |
| 0.02 | 0.186 |
| 0.03 | 0.287 |
| 0.04 | 0.363 |
| 0.05 | 0.425 |
| 0.06 | 0.478 |
| 0.07 | 0.524 |
| 0.08 | 0.566 |
| 0.09 | 0.604 |
| 0.1 | 0.638 |
| 0.15 | 0.782 |
| 0.2 | 0.896 |
| 0.25 | 0.994 |
| 0.3 | 1.082 |
| 0.35 | 1.164 |
| 0.4 | 1.241 |
| 0.45 | 1.316 |
| 0.5 | 1.39 |
| 0.55 | 1.463 |
| 0.6 | 1.538 |
| 0.65 | 1.615 |
| 0.7 | 1.697 |
| 0.75 | 1.785 |
| 0.8 | 1.883 |
| 0.85 | 1.997 |
| 0.9 | 2.141 |
| 0.91 | 2.175 |
| 0.92 | 2.213 |
| 0.93 | 2.255 |
| 0.94 | 2.301 |
| 0.95 | 2.354 |
| 0.96 | 2.416 |
| 0.97 | 2.492 |
| 0.98 | 2.593 |
| 0.99 | 2.753 |

*Plasmodium vivax*

| Cell Counts and Residuals | | | | | | | |
| --- | --- | --- | --- | --- | --- | --- | --- |
| PROBIT | Number | Concentration | Number of Subjects | Observed Responses | Expected Responses | Residual | Probability |
|  | 1 | 100000 | 4 | 4 | 4 | 0 | 1 |
|  | 2 | 10000 | 4 | 4 | 4 | 0 | 1 |
|  | 3 | 1000 | 4 | 4 | 4 | 0 | 1 |
|  | 4 | 100 | 4 | 4 | 4 | 0 | 1 |
|  | 5 | 10 | 4 | 4 | 4 | 0 | 1 |
|  | 6 | 1 | 4 | 2 | 1.582 | 0.418 | 0.396 |
|  | 7 | 0.1 | 4 | 0 | 0.628 | -0.628 | 0.157 |

| Probability | Estimate |
| --- | --- |
| 0.01 | -1.501 |
| 0.02 | -1.17 |
| 0.03 | -0.96 |
| 0.04 | -0.802 |
| 0.05 | -0.674 |
| 0.06 | -0.565 |
| 0.07 | -0.469 |
| 0.08 | -0.383 |
| 0.09 | -0.305 |
| 0.1 | -0.233 |
| 0.15 | 0.064 |
| 0.2 | 0.3 |
| 0.25 | 0.503 |
| 0.3 | 0.685 |
| 0.35 | 0.854 |
| 0.4 | 1.014 |
| 0.45 | 1.169 |
| 0.5 | 1.321 |
| 0.55 | 1.474 |
| 0.6 | 1.628 |
| 0.65 | 1.789 |
| 0.7 | 1.957 |
| 0.75 | 2.139 |
| 0.8 | 2.342 |
| 0.85 | 2.578 |
| 0.9 | 2.876 |
| 0.91 | 2.947 |
| 0.92 | 3.025 |
| 0.93 | 3.111 |
| 0.94 | 3.207 |
| 0.95 | 3.316 |
| 0.96 | 3.445 |
| 0.97 | 3.602 |
| 0.98 | 3.812 |
| 0.99 | 4.143 |

*Plasmodium ovale*

| Cell Counts and Residuals | | | | | | | |
| --- | --- | --- | --- | --- | --- | --- | --- |
| PROBIT | Number | Concentration | Number of Subjects | Observed Responses | Expected Responses | Residual | Probability |
|  | 1 | 100000 | 4 | 4 | 4 | 0 | 1 |
|  | 2 | 10000 | 4 | 4 | 4 | 0 | 1 |
|  | 3 | 1000 | 4 | 4 | 4 | 0 | 1 |
|  | 4 | 100 | 4 | 4 | 4 | 0 | 1 |
|  | 5 | 10 | 4 | 4 | 4 | 0 | 1 |
|  | 6 | 1 | 4 | 1 | 1.013 | -0.013 | 0.253 |
|  | 7 | 0.1 | 4 | 0 | 0.056 | -0.056 | 0.014 |

| Probability | Estimate |
| --- | --- |
| 0.01 | 0.026 |
| 0.02 | 0.186 |
| 0.03 | 0.287 |
| 0.04 | 0.363 |
| 0.05 | 0.425 |
| 0.06 | 0.478 |
| 0.07 | 0.524 |
| 0.08 | 0.566 |
| 0.09 | 0.604 |
| 0.1 | 0.638 |
| 0.15 | 0.782 |
| 0.2 | 0.896 |
| 0.25 | 0.994 |
| 0.3 | 1.082 |
| 0.35 | 1.164 |
| 0.4 | 1.241 |
| 0.45 | 1.316 |
| 0.5 | 1.39 |
| 0.55 | 1.463 |
| 0.6 | 1.538 |
| 0.65 | 1.615 |
| 0.7 | 1.697 |
| 0.75 | 1.785 |
| 0.8 | 1.883 |
| 0.85 | 1.997 |
| 0.9 | 2.141 |
| 0.91 | 2.175 |
| 0.92 | 2.213 |
| 0.93 | 2.255 |
| 0.94 | 2.301 |
| 0.95 | 2.354 |
| 0.96 | 2.416 |
| 0.97 | 2.492 |
| 0.98 | 2.593 |
| 0.99 | 2.753 |

*Plasmodium malariae*

| Cell Counts and Residuals | | | | | | | |
| --- | --- | --- | --- | --- | --- | --- | --- |
| PROBIT | Number | Concentration | Number of Subjects | Observed Responses | Expected Responses | Residual | Probability |
|  | 1 | 100000 | 4 | 4 | 4 | 0 | 1 |
|  | 2 | 10000 | 4 | 4 | 4 | 0 | 1 |
|  | 3 | 1000 | 4 | 4 | 4 | 0 | 1 |
|  | 4 | 100 | 4 | 4 | 4 | 0 | 1 |
|  | 5 | 10 | 4 | 4 | 4 | 0 | 1 |
|  | 6 | 1 | 4 | 2 | 1.582 | 0.418 | 0.396 |
|  | 7 | 0.1 | 4 | 0 | 0.628 | -0.628 | 0.157 |

| Probability | Estimate |
| --- | --- |
| 0.01 | -1.501 |
| 0.02 | -1.17 |
| 0.03 | -0.96 |
| 0.04 | -0.802 |
| 0.05 | -0.674 |
| 0.06 | -0.565 |
| 0.07 | -0.469 |
| 0.08 | -0.383 |
| 0.09 | -0.305 |
| 0.1 | -0.233 |
| 0.15 | 0.064 |
| 0.2 | 0.3 |
| 0.25 | 0.503 |
| 0.3 | 0.685 |
| 0.35 | 0.854 |
| 0.4 | 1.014 |
| 0.45 | 1.169 |
| 0.5 | 1.321 |
| 0.55 | 1.474 |
| 0.6 | 1.628 |
| 0.65 | 1.789 |
| 0.7 | 1.957 |
| 0.75 | 2.139 |
| 0.8 | 2.342 |
| 0.85 | 2.578 |
| 0.9 | 2.876 |
| 0.91 | 2.947 |
| 0.92 | 3.025 |
| 0.93 | 3.111 |
| 0.94 | 3.207 |
| 0.95 | 3.316 |
| 0.96 | 3.445 |
| 0.97 | 3.602 |
| 0.98 | 3.812 |
| 0.99 | 4.143 |

*Plasmodium knowlesi*

| Cell Counts and Residuals | | | | | | | |
| --- | --- | --- | --- | --- | --- | --- | --- |
| PROBIT | Number | Concentration | Number of Subjects | Observed Responses | Expected Responses | Residual | Probability |
|  | 1 | 100000 | 4 | 4 | 4 | 0 | 1 |
|  | 2 | 10000 | 4 | 4 | 4 | 0 | 1 |
|  | 3 | 1000 | 4 | 4 | 4 | 0 | 1 |
|  | 4 | 100 | 4 | 4 | 4 | 0 | 1 |
|  | 5 | 10 | 4 | 4 | 4 | 0 | 1 |
|  | 6 | 1 | 4 | 2 | 1.582 | 0.418 | 0.396 |
|  | 7 | 0.1 | 4 | 0 | 0.628 | -0.628 | 0.157 |

| Probability | Estimate |
| --- | --- |
| 0.01 | -1.501 |
| 0.02 | -1.17 |
| 0.03 | -0.96 |
| 0.04 | -0.802 |
| 0.05 | -0.674 |
| 0.06 | -0.565 |
| 0.07 | -0.469 |
| 0.08 | -0.383 |
| 0.09 | -0.305 |
| 0.1 | -0.233 |
| 0.15 | 0.064 |
| 0.2 | 0.3 |
| 0.25 | 0.503 |
| 0.3 | 0.685 |
| 0.35 | 0.854 |
| 0.4 | 1.014 |
| 0.45 | 1.169 |
| 0.5 | 1.321 |
| 0.55 | 1.474 |
| 0.6 | 1.628 |
| 0.65 | 1.789 |
| 0.7 | 1.957 |
| 0.75 | 2.139 |
| 0.8 | 2.342 |
| 0.85 | 2.578 |
| 0.9 | 2.876 |
| 0.91 | 2.947 |
| 0.92 | 3.025 |
| 0.93 | 3.111 |
| 0.94 | 3.207 |
| 0.95 | 3.316 |
| 0.96 | 3.445 |
| 0.97 | 3.602 |
| 0.98 | 3.812 |
| 0.99 | 4.143 |

Genus *Plasmodium*

| Cell Counts and Residuals | | | | | | | |
| --- | --- | --- | --- | --- | --- | --- | --- |
| PROBIT | Number | Concentration | Number of Subjects | Observed Responses | Expected Responses | Residual | Probability |
|  | 1 | 100000 | 4 | 4 | 4 | 0 | 1 |
|  | 2 | 10000 | 4 | 4 | 4 | 0 | 1 |
|  | 3 | 1000 | 4 | 4 | 4 | 0 | 1 |
|  | 4 | 100 | 4 | 4 | 4 | 0 | 1 |
|  | 5 | 10 | 4 | 4 | 4 | 0 | 1 |
|  | 6 | 1 | 4 | 1 | 1.013 | -0.013 | 0.253 |
|  | 7 | 0.1 | 4 | 0 | 0.056 | -0.056 | 0.014 |

| Probability | Estimate |
| --- | --- |
| 0.01 | 0.026 |
| 0.02 | 0.186 |
| 0.03 | 0.287 |
| 0.04 | 0.363 |
| 0.05 | 0.425 |
| 0.06 | 0.478 |
| 0.07 | 0.524 |
| 0.08 | 0.566 |
| 0.09 | 0.604 |
| 0.1 | 0.638 |
| 0.15 | 0.782 |
| 0.2 | 0.896 |
| 0.25 | 0.994 |
| 0.3 | 1.082 |
| 0.35 | 1.164 |
| 0.4 | 1.241 |
| 0.45 | 1.316 |
| 0.5 | 1.39 |
| 0.55 | 1.463 |
| 0.6 | 1.538 |
| 0.65 | 1.615 |
| 0.7 | 1.697 |
| 0.75 | 1.785 |
| 0.8 | 1.883 |
| 0.85 | 1.997 |
| 0.9 | 2.141 |
| 0.91 | 2.175 |
| 0.92 | 2.213 |
| 0.93 | 2.255 |
| 0.94 | 2.301 |
| 0.95 | 2.354 |
| 0.96 | 2.416 |
| 0.97 | 2.492 |
| 0.98 | 2.593 |
| 0.99 | 2.753 |

**Supplementary file 3. Hexaplex PCR-HRM assay sensitivity and specificity.**

| No. | Samples | Reported using molecular methods | Hexaplex PCR-HRM | | | | | | | |
| --- | --- | --- | --- | --- | --- | --- | --- | --- | --- | --- |
|  |  |  | *P. falciparum* | *P. vivax* | *P. ovale* | *P. malariae* | *P. knowlesi* | genus *Plasmodium* | Internal control | Results |
| 1 | PF0136 | *P. falciparum* | Positive | Negative | Negative | Negative | Negative | Positive | Positive | *P. falciparum* |
| 2 | PF0159 | *P. falciparum* | Positive | Negative | Negative | Negative | Negative | Positive | Positive | *P. falciparum* |
| 3 | PF0160 | *P. falciparum* | Positive | Negative | Negative | Negative | Negative | Positive | Positive | *P. falciparum* |
| 4 | PF0165 | *P. falciparum* | Positive | Negative | Negative | Negative | Negative | Positive | Positive | *P. falciparum* |
| 5 | PF0172 | *P. falciparum* | Positive | Negative | Negative | Negative | Negative | Positive | Positive | *P. falciparum* |
| 6 | PF0187 | *P. falciparum* | Positive | Negative | Negative | Negative | Negative | Positive | Positive | *P. falciparum* |
| 7 | PF0202 | *P. falciparum* | Positive | Negative | Negative | Negative | Negative | Positive | Positive | *P. falciparum* |
| 8 | PF0205 | *P. falciparum* | Positive | Negative | Negative | Negative | Negative | Positive | Positive | *P. falciparum* |
| 9 | PF0216 | *P. falciparum* | Positive | Negative | Negative | Negative | Negative | Positive | Positive | *P. falciparum* |
| 10 | PF0293 | *P. falciparum* | Positive | Negative | Negative | Negative | Negative | Positive | Positive | *P. falciparum* |
| 11 | PF0239 | *P. falciparum* | Positive | Negative | Negative | Negative | Negative | Positive | Positive | *P. falciparum* |
| 12 | PF0244 | *P. falciparum* | Positive | Negative | Negative | Negative | Negative | Positive | Positive | *P. falciparum* |
| 13 | PF0248 | *P. falciparum* | Positive | Negative | Negative | Negative | Negative | Positive | Positive | *P. falciparum* |
| 14 | PF0252 | *P. falciparum* | Positive | Negative | Negative | Negative | Negative | Positive | Positive | *P. falciparum* |
| 15 | PF0253 | *P. falciparum* | Positive | Negative | Negative | Negative | Negative | Positive | Positive | *P. falciparum* |
| 16 | PF0279 | *P. falciparum* | Positive | Negative | Negative | Negative | Negative | Positive | Positive | *P. falciparum* |
| 17 | PF0281 | *P. falciparum* | Positive | Negative | Negative | Negative | Negative | Positive | Positive | *P. falciparum* |
| 18 | PF0282 | *P. falciparum* | Positive | Negative | Negative | Negative | Negative | Positive | Positive | *P. falciparum* |
| 19 | PF0283 | *P. falciparum* | Positive | Negative | Negative | Negative | Negative | Positive | Positive | *P. falciparum* |
| 20 | PF0155 | *P. falciparum* | Positive | Negative | Negative | Negative | Negative | Positive | Positive | *P. falciparum* |
| 21 | PV206 | *P.vivax* | Negative | Positive | Negative | Negative | Negative | Positive | Positive | *P.vivax* |
| 22 | PV207 | *P.vivax* | Negative | Positive | Negative | Negative | Negative | Positive | Positive | *P.vivax* |
| 23 | PV209 | *P.vivax* | Negative | Positive | Negative | Negative | Negative | Positive | Positive | *P.vivax* |
| 24 | PV218 | *P.vivax* | Negative | Positive | Negative | Negative | Negative | Positive | Positive | *P.vivax* |
| 25 | PV131 | *P.vivax* | Negative | Positive | Negative | Negative | Negative | Positive | Positive | *P.vivax* |
| 26 | PV138 | *P.vivax* | Negative | Positive | Negative | Negative | Negative | Positive | Positive | *P.vivax* |
| 27 | PV140 | *P.vivax* | Negative | Positive | Negative | Negative | Negative | Positive | Positive | *P.vivax* |
| 28 | PV150 | *P.vivax* | Negative | Positive | Negative | Negative | Negative | Positive | Positive | *P.vivax* |
| 29 | PV153 | *P.vivax* | Negative | Positive | Negative | Negative | Negative | Positive | Positive | *P.vivax* |
| 30 | PV154 | *P.vivax* | Negative | Positive | Negative | Negative | Negative | Positive | Positive | *P.vivax* |
| 31 | PV166 | *P.vivax* | Negative | Positive | Negative | Negative | Negative | Positive | Positive | *P.vivax* |
| 32 | PV167 | *P.vivax* | Negative | Positive | Negative | Negative | Negative | Positive | Positive | *P.vivax* |
| 33 | PV168 | *P.vivax* | Negative | Positive | Negative | Negative | Negative | Positive | Positive | *P.vivax* |
| 34 | PV169 | *P.vivax* | Negative | Positive | Negative | Negative | Negative | Positive | Positive | *P.vivax* |
| 35 | PV170 | *P.vivax* | Negative | Positive | Negative | Negative | Negative | Positive | Positive | *P.vivax* |
| 36 | PV171 | *P.vivax* | Negative | Positive | Negative | Negative | Negative | Positive | Positive | *P.vivax* |
| 37 | PV173 | *P.vivax* | Negative | Positive | Negative | Negative | Negative | Positive | Positive | *P.vivax* |
| 38 | PV174 | *P.vivax* | Negative | Positive | Negative | Negative | Negative | Positive | Positive | *P.vivax* |
| 39 | PV176 | *P.vivax* | Negative | Positive | Negative | Negative | Negative | Positive | Positive | *P.vivax* |
| 40 | PV186 | *P.vivax* | Negative | Positive | Negative | Negative | Negative | Positive | Positive | *P.vivax* |
| 41 | PV177 | *P.vivax* | Negative | Positive | Negative | Negative | Negative | Positive | Positive | *P.vivax* |
| 42 | PV178 | *P.vivax* | Negative | Positive | Negative | Negative | Negative | Positive | Positive | *P.vivax* |
| 43 | PV182 | *P.vivax* | Negative | Positive | Negative | Negative | Negative | Positive | Positive | *P.vivax* |
| 44 | PV183 | *P.vivax* | Negative | Positive | Negative | Negative | Negative | Positive | Positive | *P.vivax* |
| 45 | PV184 | *P.vivax* | Negative | Positive | Negative | Negative | Negative | Positive | Positive | *P.vivax* |
| 46 | PV192 | *P.vivax* | Negative | Positive | Negative | Negative | Negative | Positive | Positive | *P.vivax* |
| 47 | PV194 | *P.vivax* | Negative | Positive | Negative | Negative | Negative | Positive | Positive | *P.vivax* |
| 48 | PV195 | *P.vivax* | Negative | Positive | Negative | Negative | Negative | Positive | Positive | *P.vivax* |
| 49 | PV197 | *P.vivax* | Negative | Positive | Negative | Negative | Negative | Positive | Positive | *P.vivax* |
| 50 | PV200 | *P.vivax* | Negative | Positive | Negative | Negative | Negative | Positive | Positive | *P.vivax* |
| 51 | POW2 | *P. ovale* | Negative | Negative | Positive | Negative | Negative | Negative | Positive | *Negative* |
| 52 | POW1 | *P. ovale* | Negative | Negative | Positive | Negative | Negative | Negative | Positive | *Negative* |
| 53 | POW3 | *P. ovale* | Negative | Negative | Positive | Negative | Negative | Positive | Positive | *P. ovale* |
| 54 | POW4 | *P. ovale* | Negative | Negative | Positive | Negative | Negative | Negative | Positive | *Negative* |
| 55 | POW5 | *P. ovale* | Negative | Negative | Positive | Negative | Negative | Positive | Positive | *P. ovale* |
| 56 | POC1 | *P. ovale* | Negative | Negative | Positive | Negative | Negative | Positive | Positive | *P. ovale* |
| 57 | POC2 | *P. ovale* | Negative | Negative | Positive | Negative | Negative | Positive | Positive | *P. ovale* |
| 58 | POC3 | *P. ovale* | Negative | Negative | Positive | Negative | Negative | Positive | Positive | *P. ovale* |
| 59 | POC4 | *P. ovale* | Negative | Negative | Positive | Negative | Negative | Positive | Positive | *P. ovale* |
| 60 | POC5 | *P. ovale* | Negative | Negative | Positive | Negative | Negative | Positive | Positive | *P. ovale* |
| 61 | PM5 | *P. malariae* | Negative | Negative | Negative | Positive | Negative | Positive | Positive | *P. malariae* |
| 62 | PM2 | *P. malariae* | Negative | Negative | Negative | Positive | Negative | Positive | Positive | *P. malariae* |
| 63 | PM9 | *P. malariae* | Negative | Negative | Negative | Positive | Negative | Positive | Positive | *P. malariae* |
| 64 | PM1 | *P. malariae* | Negative | Negative | Negative | Positive | Negative | Positive | Positive | *P. malariae* |
| 65 | PM3 | *P. malariae* | Negative | Negative | Positive | Negative | Negative | Negative | Positive | *Negative* |
| 66 | PM4 | *P. malariae* | Negative | Negative | Positive | Negative | Negative | Positive | Positive | *P.ovale* |
| 67 | PM6 | *P. malariae* | Negative | Negative | Negative | Positive | Negative | Negative | Positive | *Negative* |
| 68 | PM7 | *P. malariae* | Negative | Negative | Negative | Positive | Negative | Negative | Positive | *Negative* |
| 69 | PM8 | *P. malariae* | Negative | Negative | Negative | Positive | Negative | Positive | Positive | *P. malariae* |
| 70 | PM10 | *P. malariae* | Negative | Negative | Positive | Negative | Negative | Negative | Positive | *Negative* |
| 71 | PK001 | *P. knowlesi* | Negative | Negative | Negative | Negative | Positive | Positive | Positive | *P. knowlesi* |
| 72 | PK002 | *P. knowlesi* | Negative | Negative | Negative | Negative | Positive | Positive | Positive | *P. knowlesi* |
| 73 | PK003 | *P. knowlesi* | Negative | Negative | Negative | Negative | Positive | Positive | Positive | *P. knowlesi* |
| 74 | PK004 | *P. knowlesi* | Negative | Negative | Negative | Negative | Positive | Positive | Positive | *P. knowlesi* |
| 75 | PK005 | *P. knowlesi* | Negative | Negative | Negative | Negative | Positive | Positive | Positive | *P. knowlesi* |
| 76 | PK006 | *P. knowlesi* | Negative | Negative | Negative | Negative | Positive | Positive | Positive | *P. knowlesi* |
| 77 | PK009 | *P. knowlesi* | Negative | Negative | Negative | Negative | Positive | Positive | Positive | *P. knowlesi* |
| 78 | PK010 | *P. knowlesi* | Negative | Negative | Negative | Negative | Positive | Positive | Positive | *P. knowlesi* |
| 79 | PK011 | *P. knowlesi* | Negative | Negative | Negative | Negative | Positive | Positive | Positive | *P. knowlesi* |
| 80 | PK012 | *P. knowlesi* | Negative | Negative | Negative | Negative | Positive | Positive | Positive | *P. knowlesi* |
| 81 | PK013 | *P. knowlesi* | Negative | Negative | Negative | Negative | Positive | Positive | Positive | *P. knowlesi* |
| 82 | PK014 | *P. knowlesi* | Negative | Negative | Negative | Negative | Positive | Positive | Positive | *P. knowlesi* |
| 83 | PK015 | *P. knowlesi* | Negative | Negative | Negative | Negative | Positive | Positive | Positive | *P. knowlesi* |
| 84 | PK016 | *P. knowlesi* | Negative | Negative | Negative | Negative | Positive | Positive | Positive | *P. knowlesi* |
| 85 | PK017 | *P. knowlesi* | Negative | Negative | Negative | Negative | Positive | Positive | Positive | *P. knowlesi* |
| 86 | SH001 | Negative | Negative | Negative | Negative | Negative | Negative | Negative | Positive | Negative |
| 87 | SH002 | Negative | Negative | Negative | Negative | Negative | Negative | Negative | Positive | Negative |
| 88 | SH003 | Negative | Negative | Negative | Negative | Negative | Negative | Negative | Positive | Negative |
| 89 | SH004 | Negative | Negative | Negative | Negative | Negative | Negative | Negative | Positive | Negative |
| 90 | SH005 | Negative | Negative | Negative | Negative | Negative | Negative | Negative | Positive | Negative |
| 91 | SH006 | Negative | Negative | Negative | Negative | Negative | Negative | Negative | Positive | Negative |
| 92 | SH007 | Negative | Negative | Negative | Negative | Negative | Negative | Negative | Positive | Negative |
| 93 | SH008 | Negative | Negative | Negative | Negative | Negative | Negative | Negative | Positive | Negative |
| 94 | SH009 | Negative | Negative | Negative | Negative | Negative | Negative | Negative | Positive | Negative |
| 95 | SH010 | Negative | Negative | Negative | Negative | Negative | Negative | Negative | Positive | Negative |
| 96 | SH011 | Negative | Negative | Negative | Negative | Negative | Negative | Negative | Positive | Negative |
| 97 | SH012 | Negative | Negative | Negative | Negative | Negative | Negative | Negative | Positive | Negative |
| 98 | SH013 | Negative | Negative | Negative | Negative | Negative | Negative | Negative | Positive | Negative |
| 99 | SH014 | Negative | Negative | Negative | Negative | Negative | Negative | Negative | Positive | Negative |
| 100 | SH015 | Negative | Negative | Negative | Negative | Negative | Negative | Negative | Positive | Negative |
| 101 | H007 | Negative | Negative | Negative | Negative | Negative | Negative | Negative | Positive | Negative |
| 102 | H008 | Negative | Negative | Negative | Negative | Negative | Negative | Negative | Positive | Negative |
| 103 | H009 | Negative | Negative | Negative | Negative | Negative | Negative | Negative | Positive | Negative |
| 104 | H010 | Negative | Negative | Negative | Negative | Negative | Negative | Negative | Positive | Negative |
| 105 | A0018 | Negative | Negative | Positive | Negative | Negative | Negative | Positive | Positive | *P.vivax* |
| 106 | A0027 | Negative | Negative | Negative | Negative | Negative | Negative | Negative | Positive | Negative |
| 107 | A0028 | Negative | Negative | Negative | Negative | Negative | Negative | Negative | Positive | Negative |
| 108 | A0029 | Negative | Negative | Negative | Negative | Negative | Negative | Negative | Positive | Negative |
| 109 | A0030 | Negative | Negative | Negative | Negative | Negative | Negative | Negative | Positive | Negative |
| 110 | A0031 | Negative | Negative | Negative | Negative | Negative | Negative | Negative | Positive | Negative |
| 111 | A0032 | Negative | Negative | Negative | Negative | Negative | Negative | Negative | Positive | Negative |
| 112 | A0033 | Negative | Negative | Negative | Negative | Negative | Negative | Negative | Positive | Negative |
| 113 | A0034 | Negative | Negative | Negative | Negative | Negative | Negative | Negative | Positive | Negative |
| 114 | A0035 | Negative | Negative | Negative | Negative | Negative | Negative | Negative | Positive | Negative |
| 115 | A0036 | Negative | Negative | Negative | Negative | Negative | Negative | Negative | Positive | Negative |
| 116 | A0037 | Negative | Negative | Negative | Negative | Negative | Negative | Negative | Positive | Negative |
| 117 | A0038 | Negative | Negative | Negative | Negative | Negative | Negative | Negative | Positive | Negative |
| 118 | A0039 | Negative | Negative | Negative | Negative | Negative | Negative | Negative | Positive | Negative |
| 119 | A0040 | Negative | Negative | Negative | Negative | Negative | Negative | Negative | Positive | Negative |
| 120 | A0041 | Negative | Negative | Negative | Negative | Negative | Negative | Negative | Positive | Negative |
| 121 | A0042 | Negative | Negative | Negative | Negative | Negative | Negative | Negative | Positive | Negative |
| 122 | A0043 | Negative | Negative | Negative | Negative | Negative | Negative | Negative | Positive | Negative |
| 123 | A0044 | Negative | Negative | Negative | Negative | Negative | Negative | Negative | Positive | Negative |
| 124 | A0045 | Negative | Negative | Negative | Negative | Negative | Negative | Negative | Positive | Negative |
| 125 | A0046 | Negative | Negative | Negative | Negative | Negative | Negative | Negative | Positive | Negative |
| 126 | A0047 | Negative | Negative | Negative | Negative | Negative | Negative | Negative | Positive | Negative |
| 127 | A0048 | Negative | Negative | Negative | Negative | Negative | Negative | Negative | Positive | Negative |
| 128 | A0049 | Negative | Negative | Negative | Negative | Negative | Negative | Negative | Positive | Negative |
| 129 | A0092 | Negative | Negative | Negative | Negative | Negative | Negative | Negative | Positive | Negative |
| 130 | A0093 | Negative | Negative | Negative | Negative | Negative | Negative | Negative | Positive | Negative |
| 131 | A0094 | Negative | Negative | Negative | Negative | Negative | Negative | Negative | Positive | Negative |
| 132 | A0095 | Negative | Negative | Negative | Negative | Negative | Negative | Negative | Positive | Negative |
| 133 | A0096 | Negative | Negative | Negative | Negative | Negative | Negative | Negative | Positive | Negative |
| 134 | A0097 | Negative | Negative | Negative | Negative | Negative | Negative | Negative | Positive | Negative |
| 135 | A0098 | Negative | Negative | Negative | Negative | Negative | Negative | Negative | Positive | Negative |
| 136 | A0099 | Negative | Negative | Negative | Negative | Negative | Negative | Negative | Positive | Negative |
| 137 | A0100 | Negative | Negative | Negative | Negative | Negative | Negative | Negative | Positive | Negative |
| 138 | A0101 | Negative | Negative | Negative | Negative | Negative | Negative | Negative | Positive | Negative |
| 139 | A0102 | Negative | Negative | Negative | Negative | Negative | Negative | Negative | Positive | Negative |
| 140 | A0103 | Negative | Negative | Negative | Negative | Negative | Negative | Negative | Positive | Negative |
| 141 | A0104 | Negative | Negative | Negative | Negative | Negative | Negative | Negative | Positive | Negative |
| 142 | A0105 | Negative | Negative | Negative | Negative | Negative | Negative | Negative | Positive | Negative |
| 143 | A0106 | Negative | Negative | Negative | Negative | Negative | Negative | Negative | Positive | Negative |
| 144 | A0107 | Negative | Negative | Negative | Negative | Negative | Negative | Negative | Positive | Negative |
| 145 | A0108 | Negative | Negative | Negative | Negative | Negative | Negative | Negative | Positive | Negative |
| 146 | F01 | Negative | Negative | Negative | Negative | Negative | Negative | Negative | Positive | Negative |
| 147 | F02 | Negative | Negative | Negative | Negative | Negative | Negative | Negative | Positive | Negative |
| 148 | F03 | Negative | Negative | Negative | Negative | Negative | Negative | Negative | Positive | Negative |
| 149 | F04 | Negative | Negative | Negative | Negative | Negative | Negative | Negative | Positive | Negative |
| 150 | F05 | Negative | Negative | Negative | Negative | Negative | Negative | Negative | Positive | Negative |
| 151 | F06 | Negative | Negative | Negative | Negative | Negative | Negative | Negative | Positive | Negative |
| 152 | F07 | Negative | Negative | Negative | Negative | Negative | Negative | Negative | Positive | Negative |
| 153 | F08 | Negative | Negative | Negative | Negative | Negative | Negative | Negative | Positive | Negative |
| 154 | F09 | Negative | Negative | Negative | Negative | Negative | Negative | Negative | Positive | Negative |
| 155 | F10 | Negative | Negative | Negative | Negative | Negative | Negative | Negative | Positive | Negative |
| 156 | F11 | Negative | Negative | Negative | Negative | Negative | Negative | Negative | Positive | Negative |
| 157 | F12 | Negative | Negative | Negative | Negative | Negative | Negative | Negative | Positive | Negative |
| 158 | F13 | Negative | Negative | Negative | Negative | Negative | Negative | Negative | Positive | Negative |
| 159 | F14 | Negative | Negative | Negative | Negative | Negative | Negative | Negative | Positive | Negative |
| 160 | F15 | Negative | Negative | Negative | Negative | Negative | Negative | Negative | Positive | Negative |
| 161 | F16 | Negative | Negative | Negative | Negative | Negative | Negative | Negative | Positive | Negative |
| 162 | F17 | Negative | Negative | Negative | Negative | Negative | Negative | Negative | Positive | Negative |
| 163 | F18 | Negative | Negative | Negative | Negative | Negative | Negative | Negative | Positive | Negative |
| 164 | F19 | Negative | Negative | Negative | Negative | Negative | Negative | Negative | Positive | Negative |
| 165 | F20 | Negative | Negative | Negative | Negative | Negative | Negative | Negative | Positive | Negative |
| 166 | F21 | Negative | Negative | Negative | Negative | Negative | Negative | Negative | Positive | Negative |
| 167 | F22 | Negative | Negative | Negative | Negative | Negative | Negative | Negative | Positive | Negative |
| 168 | F23 | Negative | Negative | Negative | Negative | Negative | Negative | Negative | Positive | Negative |
| 169 | F24 | Negative | Negative | Negative | Negative | Negative | Negative | Negative | Positive | Negative |
| 170 | F25 | Negative | Negative | Negative | Negative | Negative | Negative | Negative | Positive | Negative |
| 171 | F26 | Negative | Negative | Negative | Negative | Negative | Negative | Negative | Positive | Negative |
| 172 | F27 | Negative | Negative | Negative | Negative | Negative | Negative | Negative | Positive | Negative |
| 173 | F28 | Negative | Negative | Negative | Negative | Negative | Negative | Negative | Positive | Negative |
| 174 | F29 | Negative | Negative | Negative | Negative | Negative | Negative | Negative | Positive | Negative |
| 175 | F30 | Negative | Negative | Negative | Negative | Negative | Negative | Negative | Positive | Negative |
| 176 | F31 | Negative | Negative | Negative | Negative | Negative | Negative | Negative | Positive | Negative |
| 177 | F32 | Negative | Negative | Negative | Negative | Negative | Negative | Negative | Positive | Negative |
| 178 | F33 | Negative | Negative | Negative | Negative | Negative | Negative | Negative | Positive | Negative |
| 179 | F34 | Negative | Negative | Negative | Negative | Negative | Negative | Negative | Positive | Negative |
| 180 | F35 | Negative | Negative | Negative | Negative | Negative | Negative | Negative | Positive | Negative |
| 181 | F36 | Negative | Negative | Negative | Negative | Negative | Negative | Negative | Positive | Negative |
| 182 | F37 | Negative | Negative | Negative | Negative | Negative | Negative | Negative | Positive | Negative |
| 183 | F38 | Negative | Negative | Negative | Negative | Negative | Negative | Negative | Positive | Negative |
| 184 | F39 | Negative | Negative | Negative | Negative | Negative | Negative | Negative | Positive | Negative |
| 185 | F40 | Negative | Negative | Negative | Negative | Negative | Negative | Negative | Positive | Negative |
| 186 | F41 | Negative | Negative | Negative | Negative | Negative | Negative | Negative | Positive | Negative |
| 187 | F42 | Negative | Negative | Negative | Negative | Negative | Negative | Negative | Positive | Negative |

Supplementary file 4. Clinical samples from Kanchanaburi, Thailand

|  | No. | Microscopic examination | Hexaplex PCR - HRM | | | | | | | |
| --- | --- | --- | --- | --- | --- | --- | --- | --- | --- | --- |
|  |  |  | *P. falciparum* | *P. vivax* | *P. ovale* | *P. malariae* | *P. knowlesi* | genus *Plasmodium* | Internal control | Results |
| 1 | 001 | *P. vivax* | Negative | Positive | Negative | Negative | Negative | Positive | Positive | *P. vivax* |
| 2 | 002 | *P. vivax* | Negative | Positive | Negative | Negative | Negative | Positive | Positive | *P. vivax* |
| 3 | 003 | *P. vivax* | Negative | Positive | Negative | Negative | Negative | Positive | Positive | *P. vivax* |
| 4 | 004 | *P. vivax* | Negative | Positive | Negative | Negative | Negative | Positive | Positive | *P. vivax* |
| 5 | 005 | *P. vivax* | Negative | Positive | Negative | Negative | Negative | Positive | Positive | *P. vivax* |
| 6 | 006 | *P. vivax* | Negative | Positive | Negative | Negative | Negative | Positive | Positive | *P. vivax* |
| 7 | 007 | *P. vivax* | Negative | Positive | Negative | Negative | Negative | Positive | Positive | *P. vivax* |
| 8 | 008 | *P. vivax* | Negative | Positive | Negative | Negative | Negative | Positive | Positive | *P. vivax* |
| 9 | 009 | *P. vivax* | Negative | Positive | Negative | Negative | Negative | Positive | Positive | *P. vivax* |
| 10 | 010 | *P. vivax* | Negative | Positive | Negative | Negative | Negative | Positive | Positive | *P. vivax* |
| 11 | 011 | *P. vivax* | Negative | Positive | Negative | Negative | Negative | Positive | Positive | *P. vivax* |
| 12 | 012 | *P. vivax* | Negative | Positive | Negative | Negative | Negative | Positive | Positive | *P. vivax* |
| 13 | 013 | *P. vivax* | Negative | Positive | Negative | Negative | Negative | Positive | Positive | *P. vivax* |
| 14 | 014 | *P. vivax* | Negative | Positive | Negative | Negative | Negative | Positive | Positive | *P. vivax* |
| 15 | 015 | *P. vivax* | Negative | Positive | Negative | Negative | Negative | Positive | Positive | *P. vivax* |
| 16 | 016 | *P. vivax* | Negative | Positive | Negative | Negative | Negative | Positive | Positive | *P. vivax* |
| 17 | 017 | *P. vivax* | Negative | Positive | Negative | Negative | Negative | Positive | Positive | *P. vivax* |
| 18 | 018 | *P. vivax* | Negative | Positive | Negative | Negative | Negative | Positive | Positive | *P. vivax* |
| 19 | 019 | *P. vivax* | Negative | Positive | Negative | Negative | Negative | Positive | Positive | *P. vivax* |
| 20 | 020 | *P. vivax* | Negative | Positive | Negative | Negative | Negative | Positive | Positive | *P. vivax* |
| 21 | 021 | *P. vivax* | Negative | Positive | Negative | Negative | Negative | Positive | Positive | *P. vivax* |
| 22 | 022 | *P. vivax* | Negative | Positive | Negative | Negative | Negative | Positive | Positive | *P. vivax* |
| 23 | 023 | *P. vivax* | Negative | Positive | Negative | Negative | Negative | Positive | Positive | *P. vivax* |
| 24 | 024 | *P. vivax* | Negative | Positive | Negative | Negative | Negative | Positive | Positive | *P. vivax* |
| 25 | 025 | *P. vivax* | Negative | Positive | Negative | Negative | Negative | Positive | Positive | *P. vivax* |
| 26 | 026 | *P. vivax* | Negative | Positive | Negative | Negative | Negative | Positive | Positive | *P. vivax* |
| 27 | 027 | *P. vivax* | Negative | Positive | Negative | Negative | Negative | Positive | Positive | *P. vivax* |
| 28 | 028 | *P. vivax* | Negative | Positive | Negative | Negative | Negative | Positive | Positive | *P. vivax* |
| 29 | 029 | *P. vivax* | Negative | Positive | Negative | Negative | Negative | Positive | Positive | *P. vivax* |
| 30 | 030 | *P. vivax* | Negative | Positive | Negative | Negative | Negative | Positive | Positive | *P. vivax* |
| 31 | 031 | *P. vivax* | Negative | Positive | Negative | Negative | Negative | Positive | Positive | *P. vivax* |
| 32 | 032 | *P. vivax* | Negative | Positive | Negative | Negative | Negative | Positive | Positive | *P. vivax* |
| 33 | 033 | *P. vivax* | Negative | Positive | Negative | Negative | Negative | Positive | Positive | *P. vivax* |
| 34 | 034 | *P. vivax* | Negative | Positive | Negative | Negative | Negative | Positive | Positive | *P. vivax* |
| 35 | 035 | *P. vivax* | Negative | Positive | Negative | Negative | Negative | Positive | Positive | *P. vivax* |
| 36 | 036 | *P. vivax* | Negative | Positive | Negative | Negative | Negative | Positive | Positive | *P. vivax* |
| 37 | 037 | *P. vivax* | Negative | Positive | Negative | Negative | Negative | Positive | Positive | *P. vivax* |
| 38 | 038 | *P. vivax* | Negative | Positive | Negative | Negative | Negative | Positive | Positive | *P. vivax* |
| 39 | 039 | *P. vivax* | Negative | Positive | Negative | Negative | Negative | Positive | Positive | *P. vivax* |
| 40 | 040 | *P. vivax* | Negative | Positive | Negative | Negative | Negative | Positive | Positive | *P. vivax* |
| 41 | 041 | *P. vivax* | Negative | Positive | Negative | Negative | Negative | Positive | Positive | *P. vivax* |
| 42 | 042 | *P. vivax* | Negative | Positive | Negative | Negative | Negative | Positive | Positive | *P. vivax* |
| 43 | 043 | *P. vivax* | Negative | Positive | Negative | Negative | Negative | Positive | Positive | *P. vivax* |
| 44 | 044 | *P. vivax* | Negative | Positive | Negative | Negative | Negative | Positive | Positive | *P. vivax* |
| 45 | 045 | *P. vivax* | Negative | Positive | Negative | Negative | Negative | Positive | Positive | *P. vivax* |
| 46 | 046 | *P. vivax* | Negative | Positive | Negative | Negative | Negative | Positive | Positive | *P. vivax* |
| 47 | 047 | *P. vivax* | Negative | Positive | Negative | Negative | Negative | Positive | Positive | *P. vivax* |
| 48 | 048 | *P. vivax* | Negative | Positive | Negative | Negative | Negative | Positive | Positive | *P. vivax* |
| 49 | 049 | *P. vivax* | Negative | Positive | Negative | Negative | Negative | Positive | Positive | *P. vivax* |
| 50 | 050 | *P. vivax* | Negative | Positive | Negative | Negative | Negative | Positive | Positive | *P. vivax* |
| 51 | 051 | *P. vivax* | Negative | Positive | Negative | Negative | Negative | Positive | Positive | *P. vivax* |
| 52 | 052 | *P. vivax* | Negative | Positive | Negative | Negative | Negative | Positive | Positive | *P. vivax* |
| 53 | 053 | *P. vivax* | Negative | Positive | Negative | Negative | Negative | Positive | Positive | *P. vivax* |
| 54 | 054 | *P. vivax* | Negative | Positive | Negative | Negative | Negative | Positive | Positive | *P. vivax* |
| 55 | 055 | *P. vivax* | Negative | Positive | Negative | Negative | Negative | Positive | Positive | *P. vivax* |
| 56 | 056 | *P. vivax* | Negative | Positive | Negative | Negative | Negative | Positive | Positive | *P. vivax* |
| 57 | 057 | *P. vivax* | Negative | Positive | Negative | Negative | Negative | Positive | Positive | *P. vivax* |
| 58 | 058 | *P. vivax* | Negative | Positive | Negative | Negative | Negative | Positive | Positive | *P. vivax* |
| 59 | 059 | *P. vivax* | Negative | Positive | Negative | Negative | Negative | Positive | Positive | *P. vivax* |
| 60 | 060 | *P. vivax* | Negative | Positive | Negative | Negative | Negative | Positive | Positive | *P. vivax* |
| 61 | 061 | *P. vivax* | Negative | Positive | Negative | Negative | Negative | Positive | Positive | *P. vivax* |
| 62 | 062 | *P. vivax* | Negative | Positive | Negative | Negative | Negative | Positive | Positive | *P. vivax* |
| 63 | 063 | *P. vivax* | Negative | Positive | Negative | Negative | Negative | Positive | Positive | *P. vivax* |
| 64 | 064 | *P. vivax* | Negative | Positive | Negative | Negative | Negative | Positive | Positive | *P. vivax* |
| 65 | 065 | *P. vivax* | Negative | Positive | Negative | Negative | Negative | Positive | Positive | *P. vivax* |
| 66 | 066 | *P. vivax* | Negative | Positive | Negative | Negative | Negative | Positive | Positive | *P. vivax* |
| 67 | 067 | *P. vivax* | Negative | Positive | Negative | Negative | Negative | Positive | Positive | *P. vivax* |
| 68 | 068 | *P. vivax* | Negative | Positive | Negative | Negative | Negative | Positive | Positive | *P. vivax* |
| 69 | 069 | *P. vivax* | Negative | Positive | Negative | Negative | Negative | Positive | Positive | *P. vivax* |
| 70 | 070 | *P. vivax* | Negative | Positive | Negative | Negative | Negative | Positive | Positive | *P. vivax* |
| 71 | 071 | *P. vivax* | Negative | Positive | Negative | Negative | Negative | Positive | Positive | *P. vivax* |
| 72 | 072 | *P. vivax* | Negative | Positive | Negative | Negative | Negative | Positive | Positive | *P. vivax* |
| 73 | 073 | *P. falciparum* | Positive | Negative | Negative | Negative | Negative | Positive | Positive | *P. falciparum* |
| 74 | 074 | *P. vivax* | Negative | Positive | Negative | Negative | Negative | Positive | Positive | *P. vivax* |
| 75 | 075 | *P. vivax* | Negative | Positive | Negative | Negative | Negative | Positive | Positive | *P. vivax* |
| 76 | 076 | *P. vivax* | Negative | Positive | Negative | Negative | Negative | Positive | Positive | *P. vivax* |
| 77 | 077 | *P. vivax* | Negative | Positive | Negative | Negative | Negative | Positive | Positive | *P. vivax* |
| 78 | 078 | *P. vivax* | Negative | Positive | Negative | Negative | Negative | Positive | Positive | *P. vivax* |
| 79 | 079 | *P. vivax* | Negative | Positive | Negative | Negative | Negative | Positive | Positive | *P. vivax* |
| 80 | 080 | *P. vivax* | Negative | Positive | Negative | Negative | Negative | Positive | Positive | *P. vivax* |
| 81 | 081 | *P. vivax* | Negative | Positive | Negative | Negative | Negative | Positive | Positive | *P. vivax* |
| 82 | 082 | *P. vivax* | Negative | Positive | Negative | Negative | Negative | Positive | Positive | *P. vivax* |
| 83 | 083 | *P. vivax* | Negative | Positive | Negative | Negative | Negative | Positive | Positive | *P. vivax* |
| 84 | 084 | *P. vivax* | Negative | Positive | Negative | Negative | Negative | Positive | Positive | *P. vivax* |
| 85 | 085 | *P. vivax* | Negative | Positive | Negative | Negative | Negative | Positive | Positive | *P. vivax* |
| 86 | 086 | *P. vivax* | Negative | Positive | Negative | Negative | Negative | Positive | Positive | *P. vivax* |
| 87 | 087 | *P. vivax* | Negative | Positive | Negative | Negative | Negative | Positive | Positive | *P. vivax* |
| 88 | 088 | *P. vivax* | Negative | Positive | Negative | Negative | Negative | Positive | Positive | *P. vivax* |
| 89 | 089 | *P. vivax* | Negative | Positive | Negative | Negative | Negative | Positive | Positive | *P. vivax* |
| 90 | 090 | *P. vivax* | Negative | Positive | Negative | Negative | Negative | Positive | Positive | *P. vivax* |
| 91 | 091 | *P. vivax* | Negative | Positive | Negative | Negative | Negative | Positive | Positive | *P. vivax* |
| 92 | 092 | *P. vivax* | Negative | Positive | Negative | Negative | Negative | Positive | Positive | *P. vivax* |
| 93 | 093 | *P. vivax* | Negative | Positive | Negative | Negative | Negative | Positive | Positive | *P. vivax* |
| 94 | 094 | *P. vivax* | Negative | Positive | Negative | Negative | Negative | Positive | Positive | *P. vivax* |
| 95 | 095 | *P. vivax* | Negative | Positive | Negative | Negative | Negative | Positive | Positive | *P. vivax* |
| 96 | 096 | *P. vivax* | Negative | Positive | Negative | Negative | Negative | Positive | Positive | *P. vivax* |
| 97 | 097 | *P. vivax* | Negative | Positive | Negative | Negative | Negative | Positive | Positive | *P. vivax* |
| 98 | 098 | *P. vivax* | Negative | Positive | Negative | Negative | Negative | Positive | Positive | *P. vivax* |
| 99 | 099 | *P. vivax* | Negative | Positive | Negative | Negative | Negative | Positive | Positive | *P. vivax* |
| 100 | 100 | *P. vivax* | Negative | Positive | Negative | Negative | Negative | Positive | Positive | *P. vivax* |
| 101 | 101 | *P. vivax* | Negative | Positive | Negative | Negative | Negative | Positive | Positive | *P. vivax* |
| 102 | 102 | *P. vivax* | Negative | Positive | Negative | Negative | Negative | Positive | Positive | *P. vivax* |
| 103 | 103 | *P. vivax* | Negative | Positive | Negative | Negative | Negative | Positive | Positive | *P. vivax* |
| 104 | 104 | *P. vivax* | Negative | Positive | Negative | Negative | Negative | Positive | Positive | *P. vivax* |
| 105 | 105 | *P. vivax* | Negative | Positive | Negative | Negative | Negative | Positive | Positive | *P. vivax* |
| 106 | 106 | *P. vivax* | Negative | Positive | Negative | Negative | Negative | Positive | Positive | *P. vivax* |
| 107 | 107 | *P. vivax* | Negative | Positive | Negative | Negative | Negative | Positive | Positive | *P. vivax* |
| 108 | 108 | *P. vivax* | Negative | Positive | Negative | Negative | Negative | Positive | Positive | *P. vivax* |
| 109 | 109 | *P. vivax* | Negative | Positive | Negative | Negative | Negative | Positive | Positive | *P. vivax* |
| 110 | 110 | *P. vivax* | Negative | Positive | Negative | Negative | Negative | Positive | Positive | *P. vivax* |
| 111 | 111 | *P. vivax* | Negative | Positive | Negative | Negative | Negative | Positive | Positive | *P. vivax* |
| 112 | 112 | *P. vivax* | Negative | Positive | Negative | Negative | Negative | Positive | Positive | *P. vivax* |
| 113 | 113 | *P. vivax* | Negative | Positive | Negative | Negative | Negative | Positive | Positive | *P. vivax* |
| 114 | 114 | *P. vivax* | Negative | Positive | Negative | Negative | Negative | Positive | Positive | *P. vivax* |
| 115 | 115 | *P. vivax* | Negative | Positive | Negative | Negative | Negative | Positive | Positive | *P. vivax* |
| 116 | 116 | *P. vivax* | Negative | Positive | Negative | Negative | Negative | Positive | Positive | *P. vivax* |
| 117 | 117 | *P. vivax* | Negative | Positive | Negative | Negative | Negative | Positive | Positive | *P. vivax* |
| 118 | 118 | *P. vivax* | Negative | Positive | Negative | Negative | Negative | Positive | Positive | *P. vivax* |
| 119 | 119 | *P. vivax* | Negative | Positive | Negative | Negative | Negative | Positive | Positive | *P. vivax* |
| 120 | 120 | *P. vivax* | Negative | Positive | Negative | Negative | Negative | Positive | Positive | *P. vivax* |

Analytical sensitivity estimated on *P.vivax* clinical samples

| Sample | Species | Parasites /ul | Hexaplex PCR | | | |
| --- | --- | --- | --- | --- | --- | --- |
|  |  |  | 1 | 2 | 3 | 4 |
| No. 003 | *P. vivax* | 2640 | Positive | Positive | Positive | Positive |
|  |  | 1320 | Positive | Positive | Positive | Positive |
|  |  | 660 | Positive | Positive | Positive | Positive |
|  |  | 330 | Positive | Positive | Positive | Positive |
|  |  | 165 | Positive | Negative | Positive | Positive |
|  |  | 82 | Positive | Positive | Negative | Negative |
|  |  | 41 | Positive | Negative | Positive | Negative |
|  |  | 20 | Negative | Negative | Negative | Positive |
|  |  | 10 | Negative | Negative | Negative | Negative |
| No. 006 | *P. vivax* | 3920 | Positive | Positive | Positive | Positive |
|  |  | 1960 | Positive | Positive | Positive | Positive |
|  |  | 980 | Positive | Positive | Positive | Positive |
|  |  | 490 | Positive | Positive | Positive | Positive |
|  |  | 245 | Positive | Positive | Positive | Positive |
|  |  | 122 | Positive | Positive | Positive | Positive |
|  |  | 61 | Positive | Negative | Negative | Positive |
|  |  | 30 | Negative | Positive | Negative | Negative |
|  |  | 15 | Negative | Negative | Negative | Negative |

Sample No. 003

| Probability | Estimate |
| --- | --- |
| 0.01 | -116.044 |
| 0.02 | -92.168 |
| 0.03 | -77.019 |
| 0.04 | -65.623 |
| 0.05 | -56.353 |
| 0.06 | -48.463 |
| 0.07 | -41.545 |
| 0.08 | -35.351 |
| 0.09 | -29.718 |
| 0.1 | -24.532 |
| 0.15 | -3.062 |
| 0.2 | 14.001 |
| 0.25 | 28.64 |
| 0.3 | 41.786 |
| 0.35 | 53.968 |
| 0.4 | 65.527 |
| 0.45 | 76.711 |
| 0.5 | 87.717 |
| 0.55 | 98.724 |
| 0.6 | 109.908 |
| 0.65 | 121.467 |
| 0.7 | 133.649 |
| 0.75 | 146.795 |
| 0.8 | 161.434 |
| 0.85 | 178.497 |
| 0.9 | 199.966 |
| 0.91 | 205.152 |
| 0.92 | 210.785 |
| 0.93 | 216.98 |
| 0.94 | 223.898 |
| 0.95 | 231.788 |
| 0.96 | 241.057 |
| 0.97 | 252.453 |
| 0.98 | 267.602 |
| 0.99 | 291.479 |

Sample No. 006

| Probability | Estimate |
| --- | --- |
| 0.01 | -2.439 |
| 0.02 | 4.527 |
| 0.03 | 8.946 |
| 0.04 | 12.271 |
| 0.05 | 14.975 |
| 0.06 | 17.277 |
| 0.07 | 19.295 |
| 0.08 | 21.102 |
| 0.09 | 22.745 |
| 0.1 | 24.258 |
| 0.15 | 30.521 |
| 0.2 | 35.499 |
| 0.25 | 39.77 |
| 0.3 | 43.605 |
| 0.35 | 47.158 |
| 0.4 | 50.531 |
| 0.45 | 53.793 |
| 0.5 | 57.004 |
| 0.55 | 60.215 |
| 0.6 | 63.478 |
| 0.65 | 66.85 |
| 0.7 | 70.404 |
| 0.75 | 74.239 |
| 0.8 | 78.509 |
| 0.85 | 83.487 |
| 0.9 | 89.75 |
| 0.91 | 91.263 |
| 0.92 | 92.907 |
| 0.93 | 94.714 |
| 0.94 | 96.732 |
| 0.95 | 99.033 |
| 0.96 | 101.738 |
| 0.97 | 105.062 |
| 0.98 | 109.482 |
| 0.99 | 116.447 |

Supplementary file 5. Multiplex HRM melting curve in derivative plot analyses of *Plasmodium*-infected and non-infected samples.

1. *Plasmodium knowlesi 2. Plasmodium malariae*

*
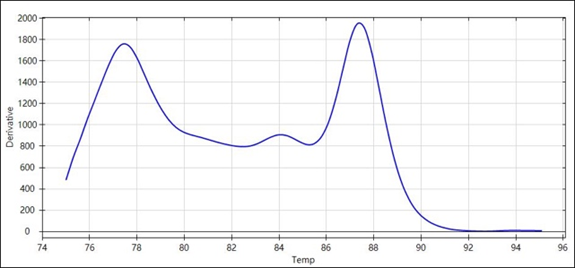

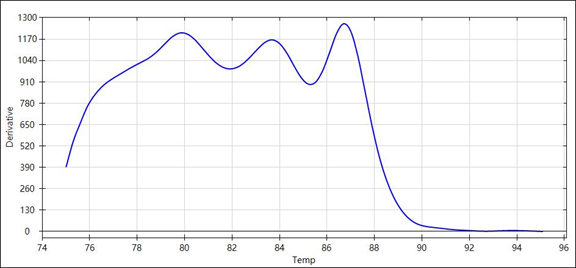
*

1. *Plasmodium ovale 4. Plasmodium vivax*

*
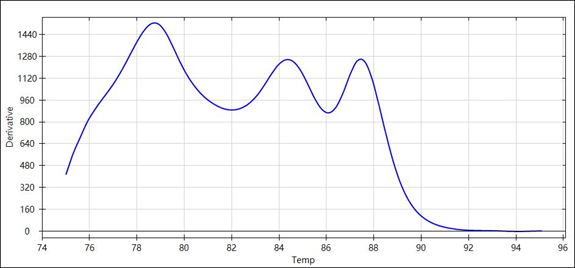

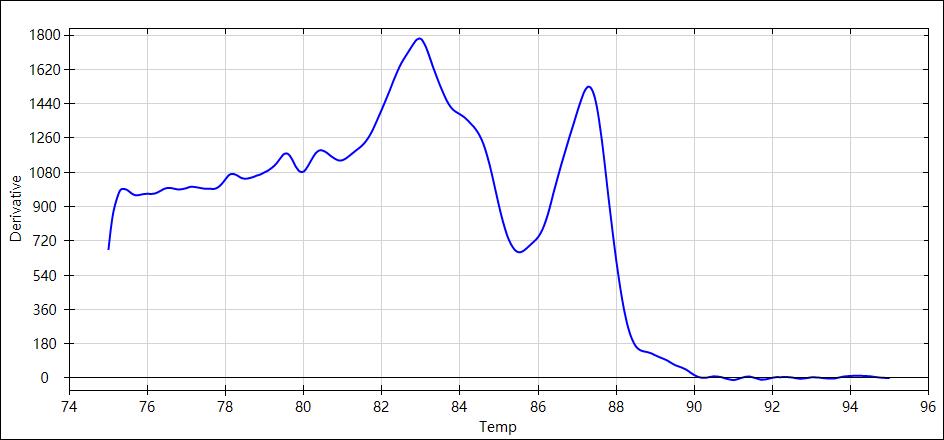
*

1. *Plasmodium falciparum 6. Non-infected Plasmodium sample*


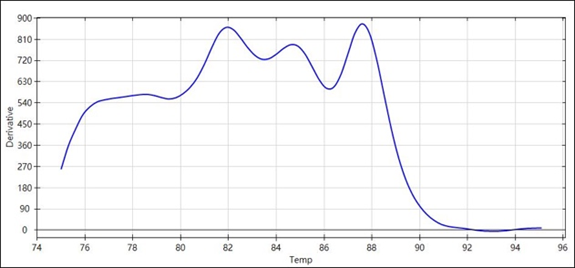

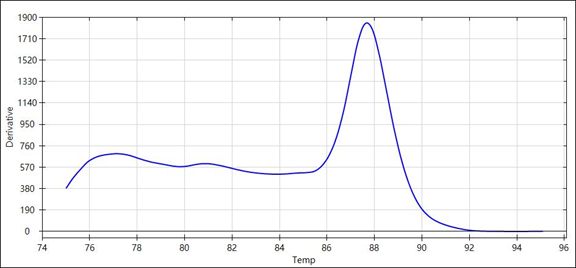

Supplement: Supplementary file 1 [file mmc1.docx]
